# Supplementary material for: A chromosome-level genome sequence of Chrysanthemum seticuspe, a model species for hexaploid cultivated chrysanthemum
Source: Commun Biol. 2021 Oct 7;4:1167. doi: 10.1038/s42003-021-02704-y (PMC8497461; doi:10.1038/s42003-021-02704-y)
Supplement: Supplementary file 2 — Supplementary information [file 42003_2021_2704_MOESM2_ESM.pdf]

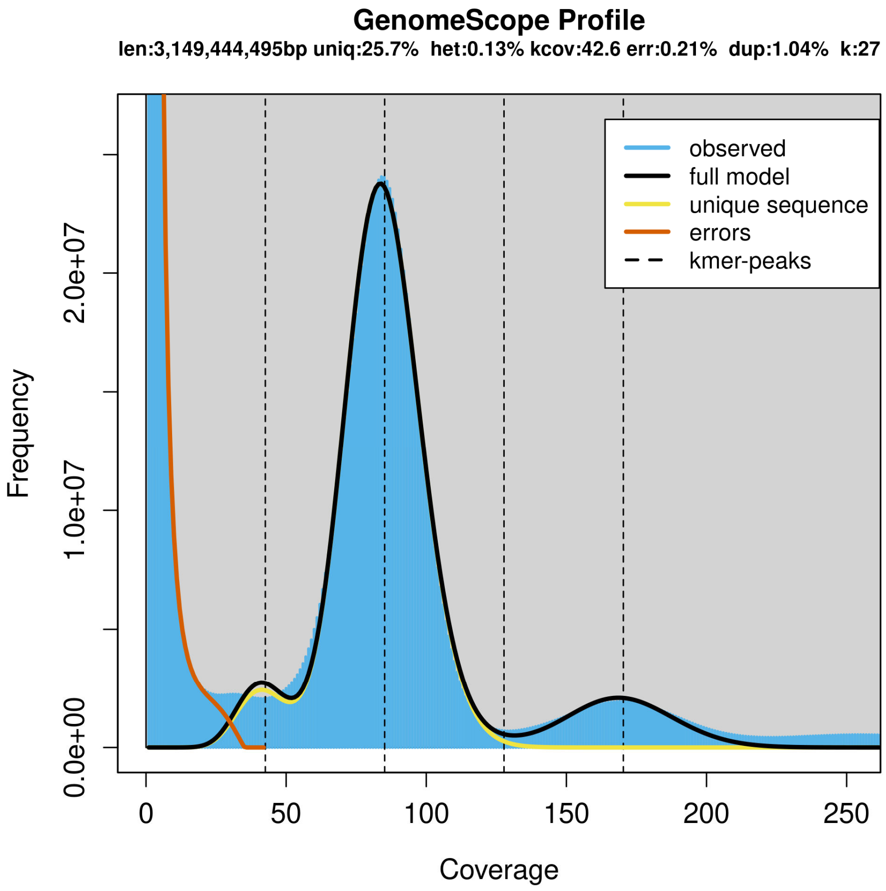

Supplementary Figure 1 | *k*-mer analysis of unassembled short-read sequences.

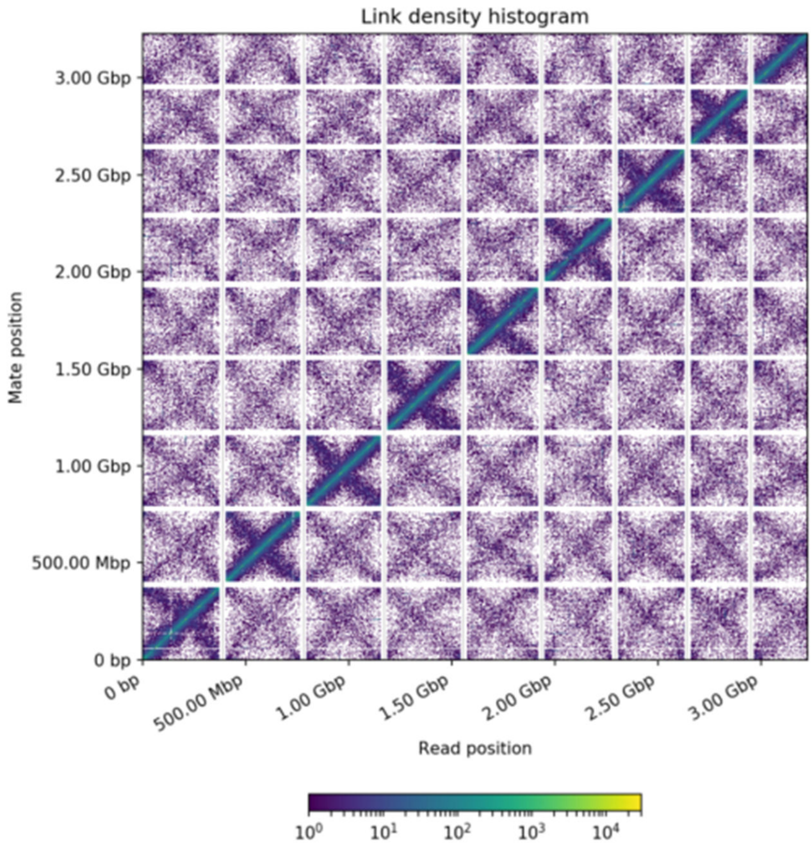

Supplementary Figure 2 | Heat map of Hi-C interactions among the nine largest scaffolds of Gojo-0 genome. The link density of paired sequencing reads in different contigs is illustrated by a color gradient.

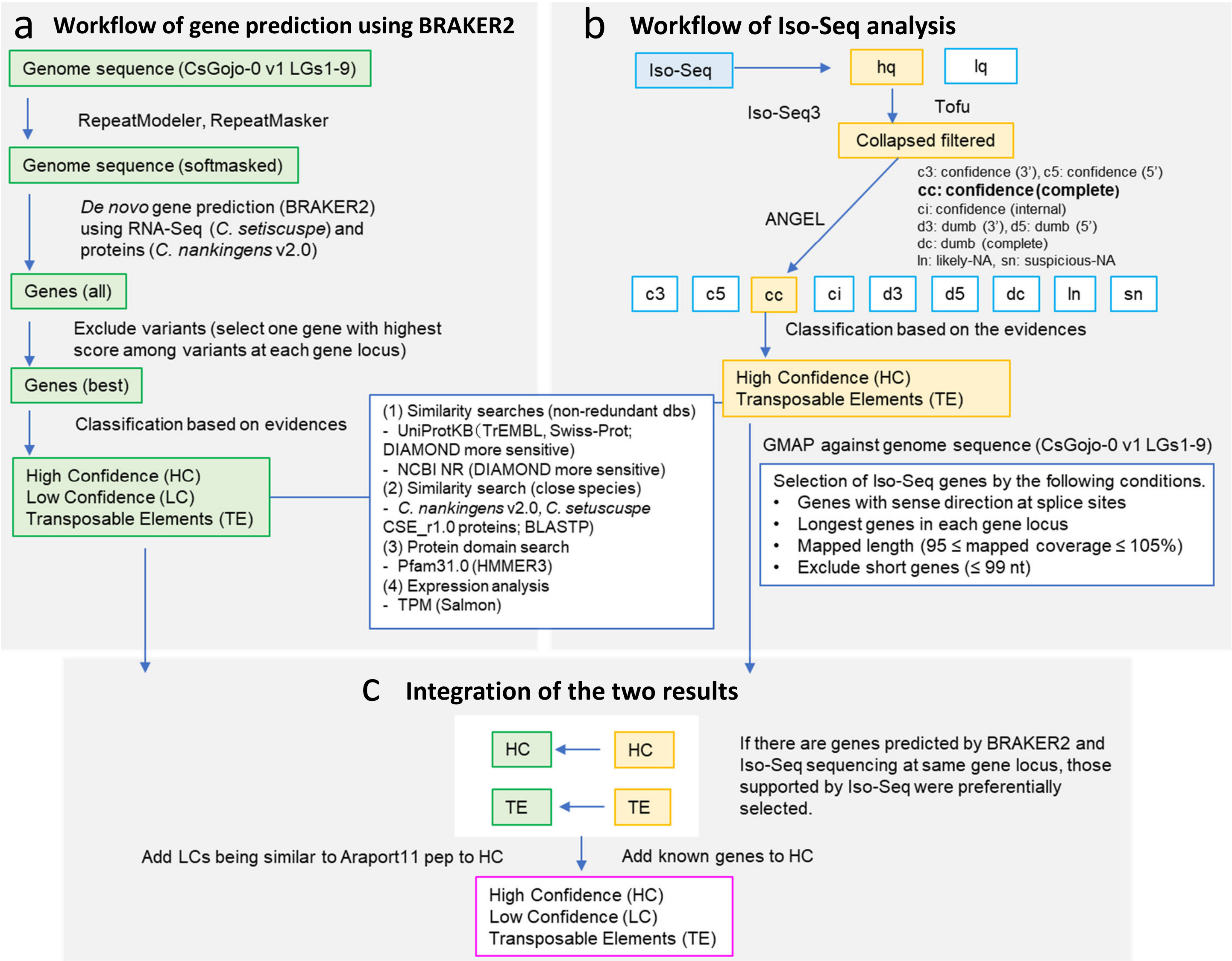

Supplementary Figure 3 | The workflow of gene prediction. a, The workflow of gene prediction using BRAKER2. **b**, The workflow of Iso-Seq analysis. **c**, Integration of genes predicted by BRAKER2 and Iso-Seq analysis.

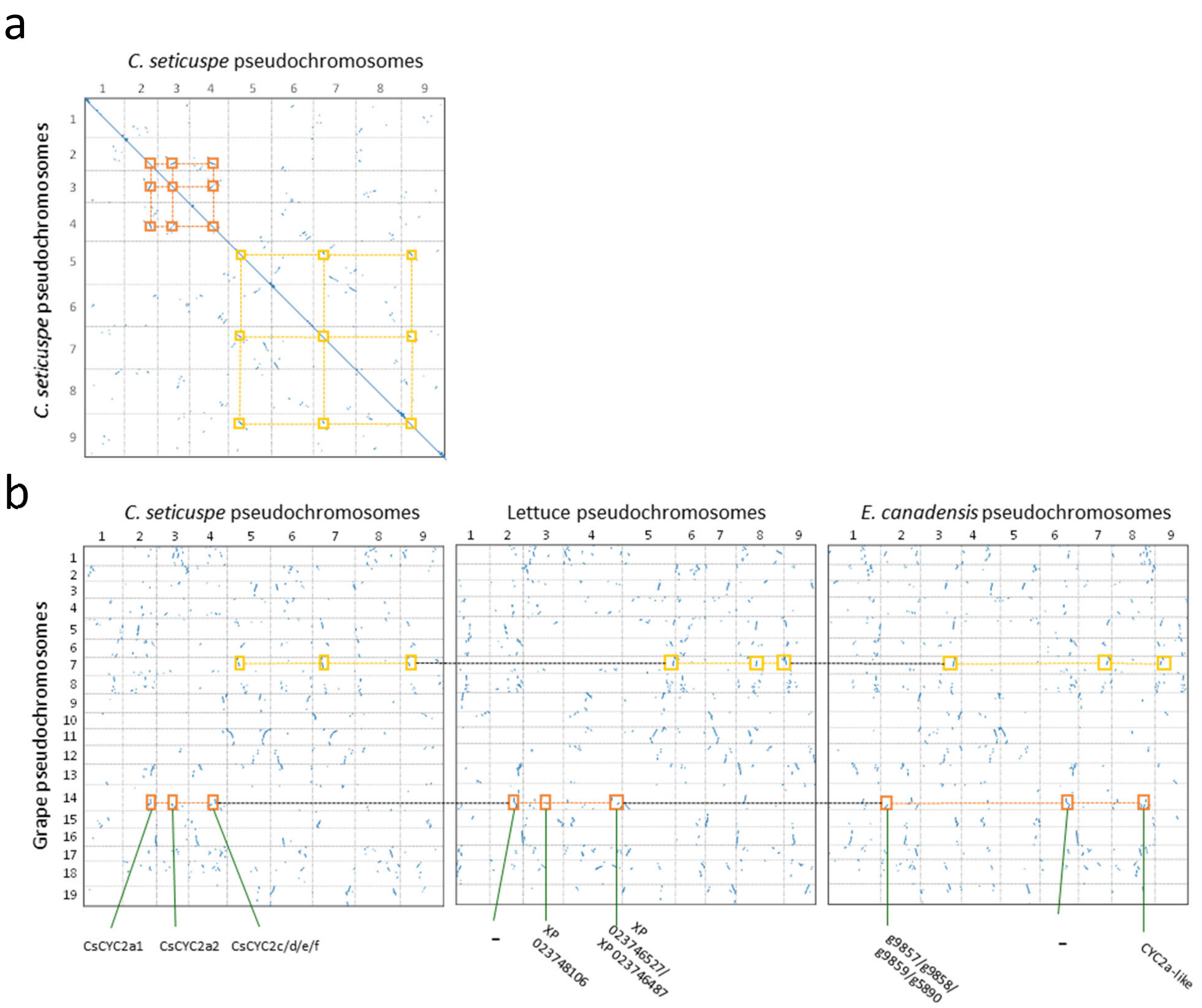

Supplementary Figure 4 | Dot plot analysis of paralogs in *Chrysanthemum seticuspe*, lettuce, and *Erigeron canadensis*. a, Dot plot analysis of *C. seticuspe* (Gojo-0) pseudochromosomes. The numbers represent the number of peudochromosomes/linkage groups. Some syntenic blocks between pseudochromosomes are boxed. The syntenic blocks corresponding to the same regions are represented by dotted lines. **b**, Dot plot analysis between grape (*V. vinifera)* and *C. seticuspe*, lettuce (*L. sativa*), and *E. canadensis*. The syntenic blocks corresponding to those indicated in **a** are boxed. The syntenic blocks corresponding to the same regions are represented by dotted lines. The *CYC2* family genes in the syntenic regions are shown in the dot plots.

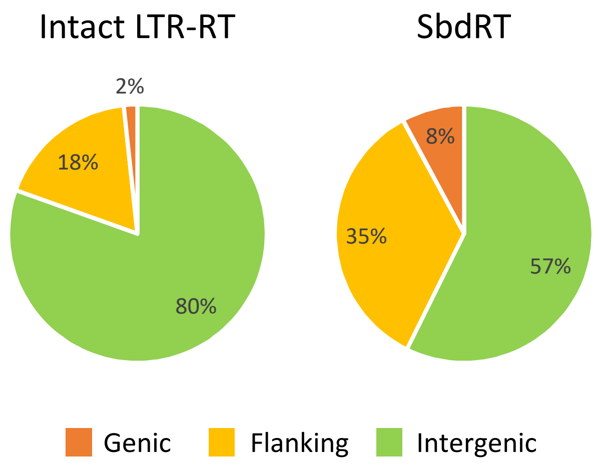

Supplementary Figure 5 | Distribution of insertion sites of SbdRT in the *C. seticuspe* genome. Genic, insertion of structural genes; flanking, insertions within 5 kb upstream or downstream of structural genes; intergenic, insertions in other regions. Intact LTR-RT represents all types of LTR-RT with LTRs at both ends. SbdRT represents all 360 SbdRT copies.

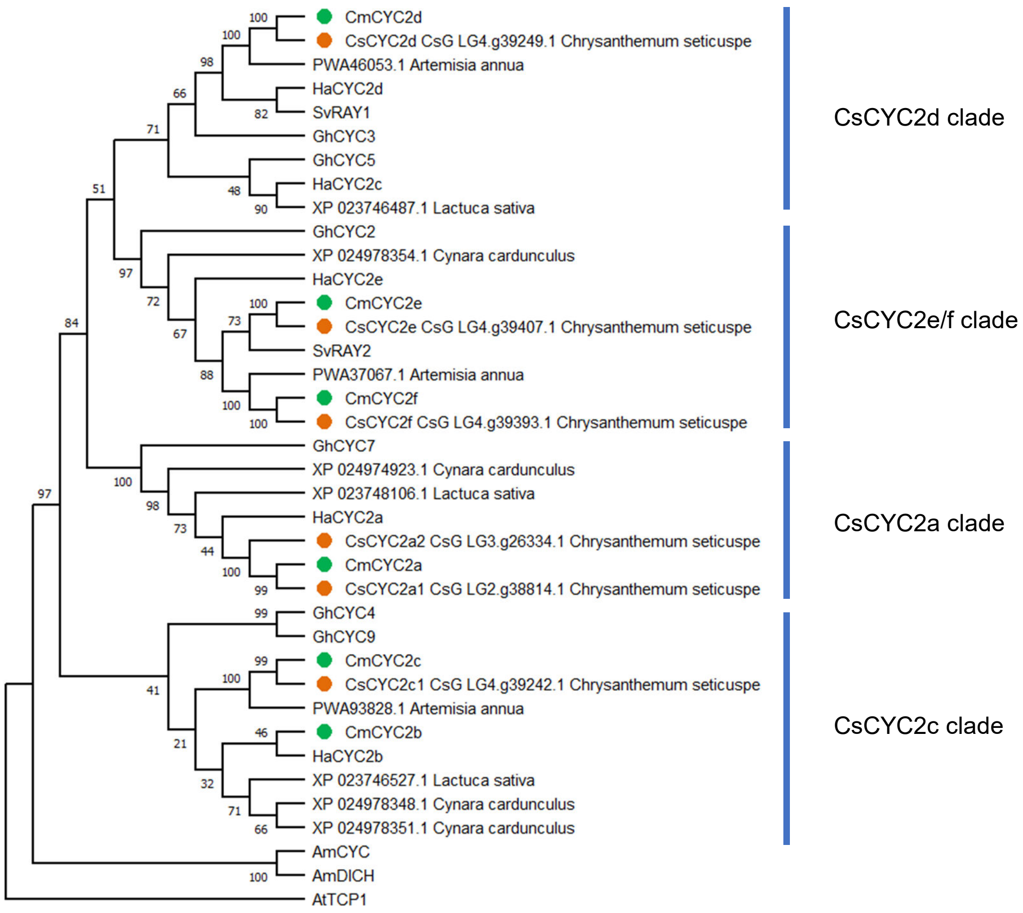

Supplementary Figure 6 | Phylogenetic tree of the *CYC2* family genes in Asteraceae. CsCYC2, CmCYC2, HaCYC2, GhCYC, and SvRAY are the *CYC2* family genes of *C. seticuspe*, *C. morifolium*, sunflower, gerbera, and *Senecio vulgaris,* respectively. The *CYC2* family genes of *C. seticuspe* and *C. morifolium* are marked with orange and green circles, respectively.

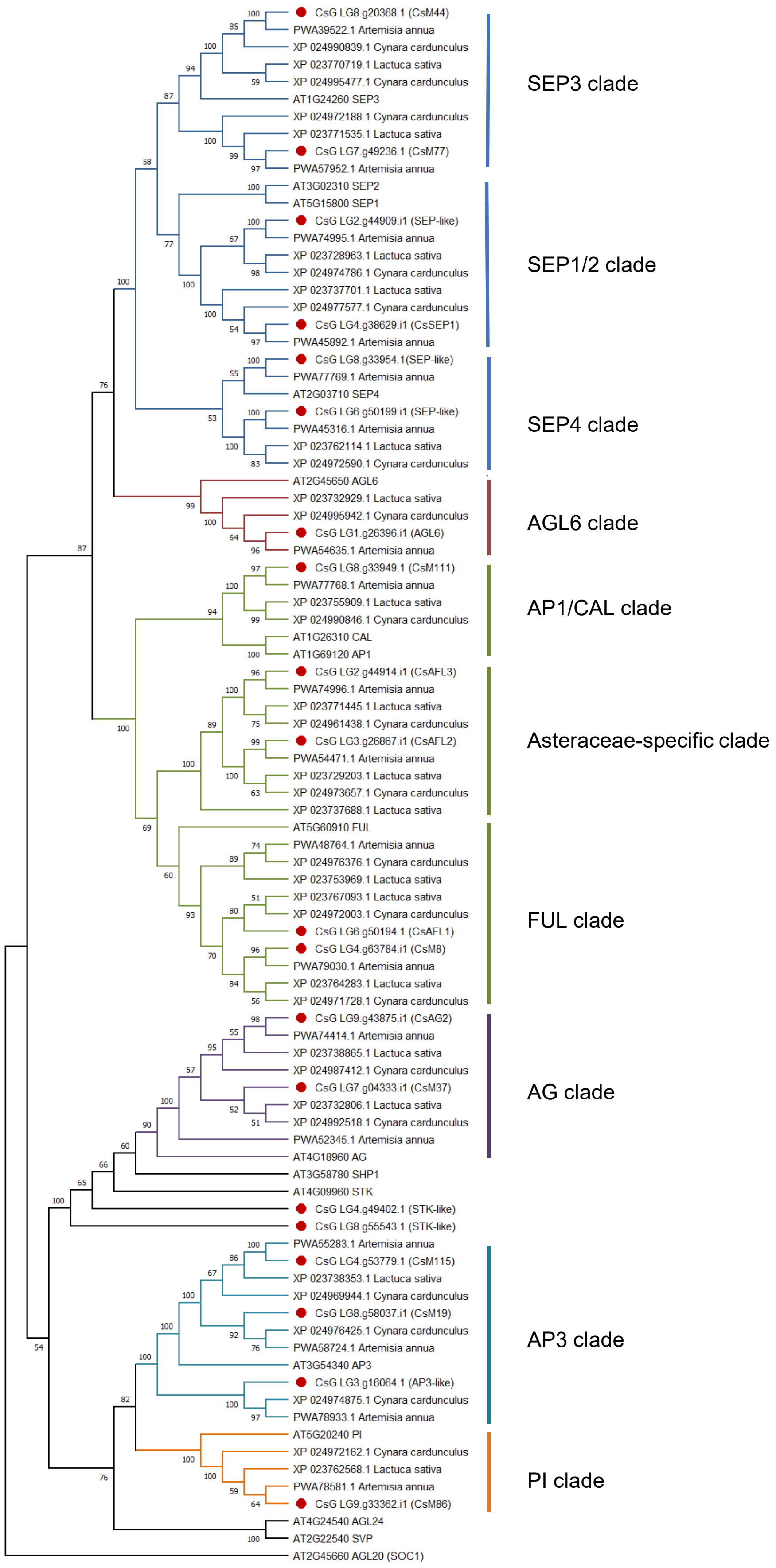

Supplementary Figure 7 | Phylogenetic tree of ABCE MADS-box genes in Asteraceae. MADS-box genes of *C. seticuspe* are marked with red circles.

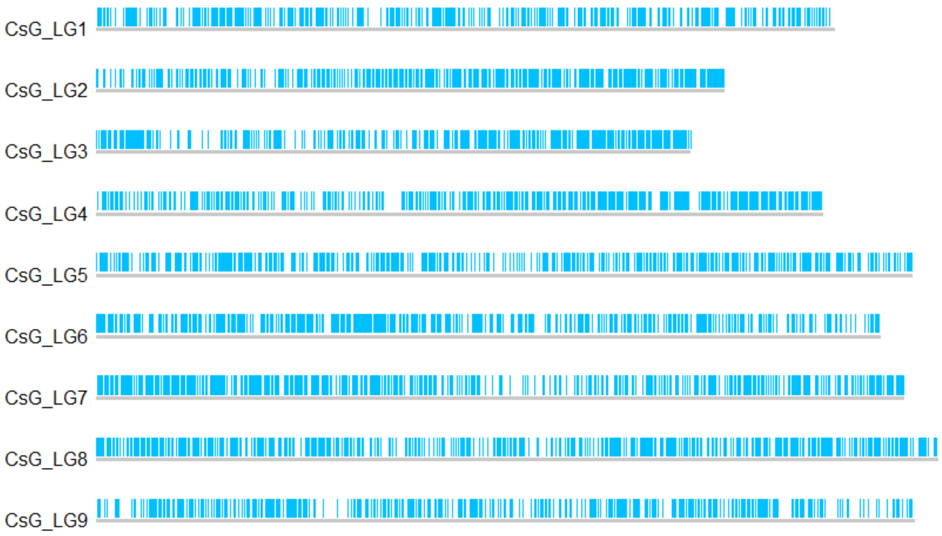

Supplementary Figure 8 | Chromosomal locations of SSR markers in the *C. seticuspe* genome. SSR markers detected in AEV02 were mapped to *C. seticuspe* pseudochromosomes.

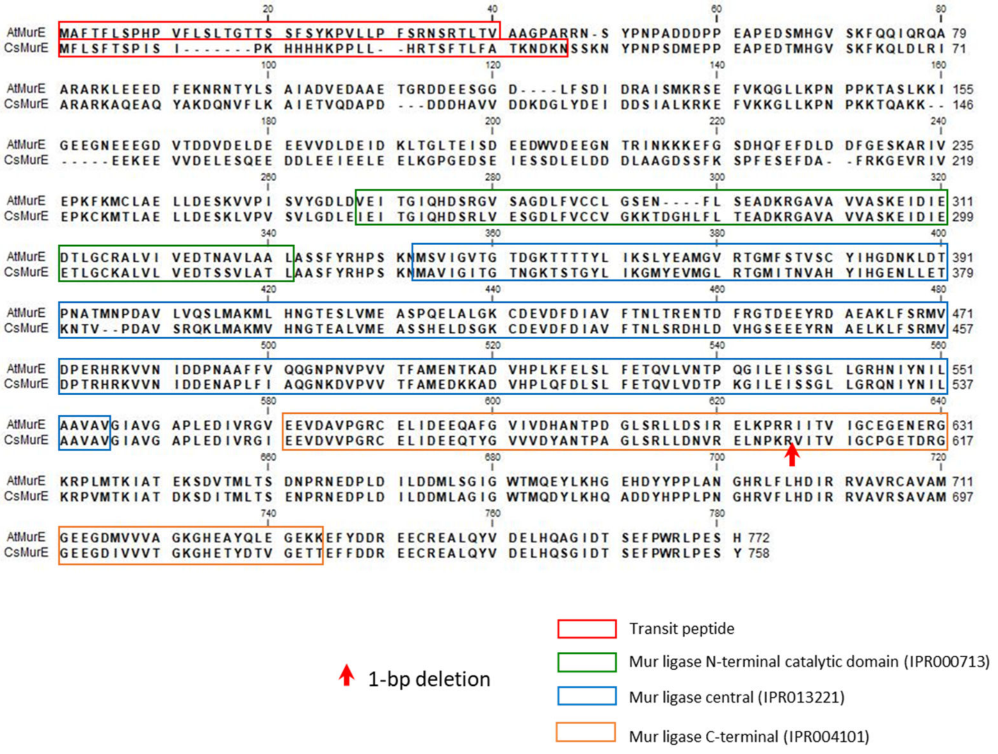

Supplementary Figure 9 | Alignment of AtMurE and CsMurE proteins. CsMurE retains chloroplast transit peptide and Mur ligase domains. The red arrow indicates the position of a single base pair deletion in *alb1*.

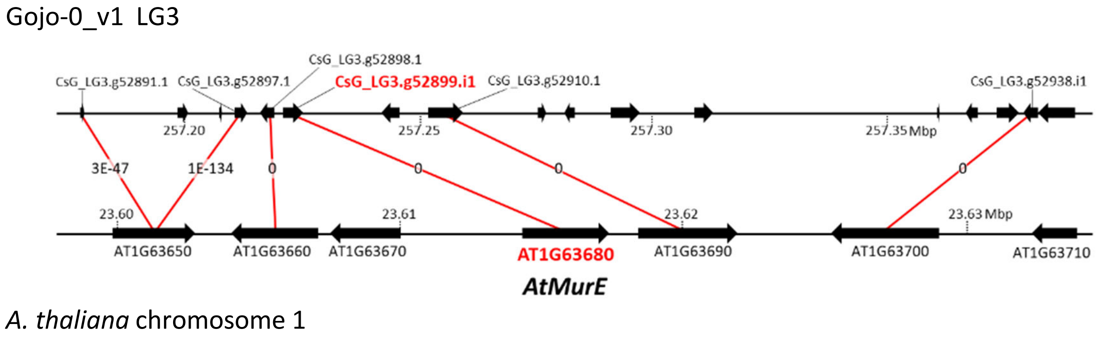

Supplementary Figure 10 | Microsynteny between the *CsMurE* and *AtMurE* regions. The predicted genes around LG3.g52899.i1 in *C. seticuspe* and *AtMurE* in *A. thaliana* are shown. The values shown on the red lines indicate e-values obtained from BLAST analysis.

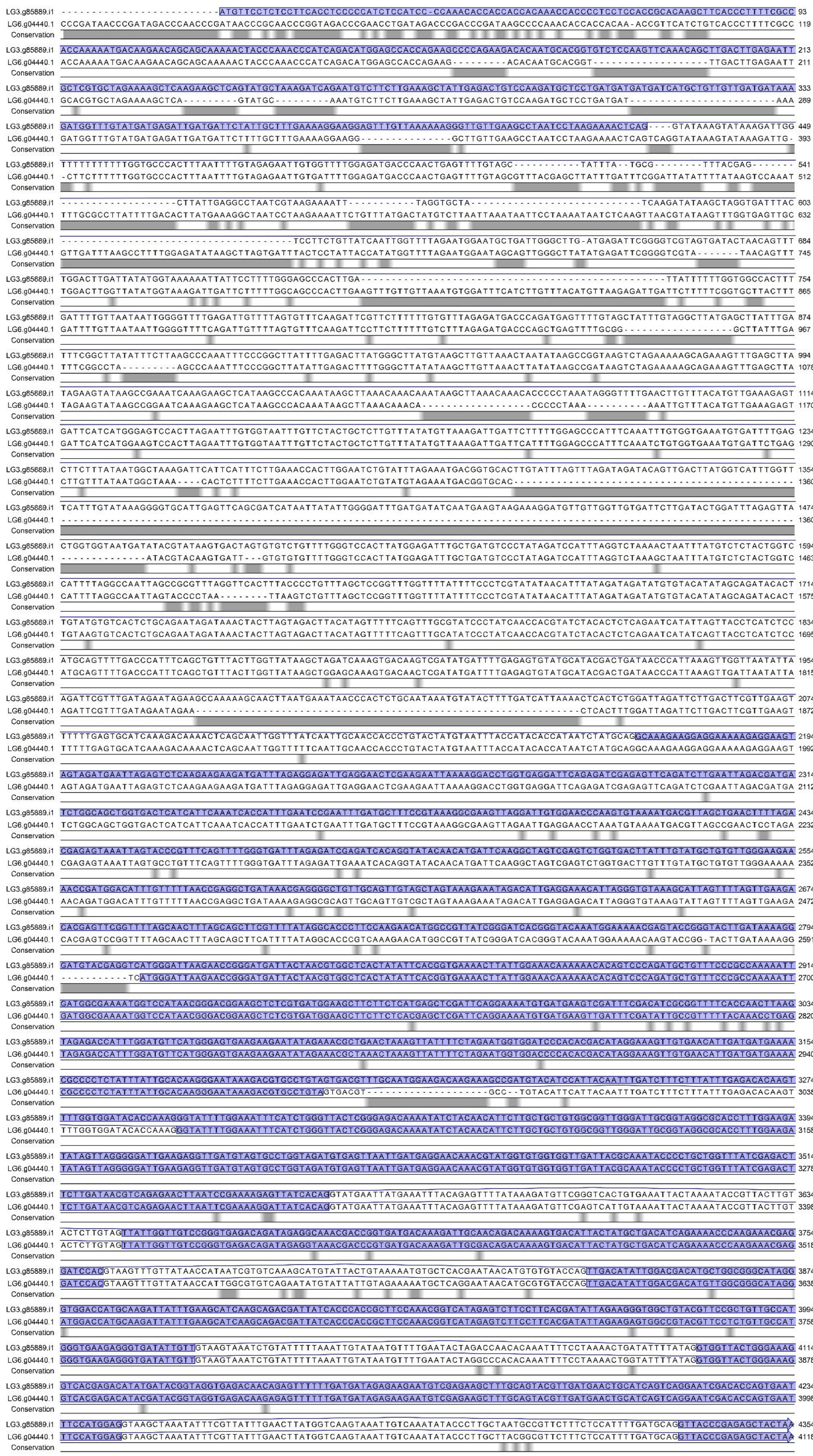

Supplementary Figure 11 | Alignment of genome sequence of LG3.g85889.i1 (*CsMurE*) and LG6.g04440.1 (pseudo gene). Purple regions are coding sequences. Gray regions show sequence differences between LG3.g85889.i1 and LG6.g04440.1. LG6.g04440.1 contains numerous deletions particularly in 5′ regions and does not encode a functional MurE.

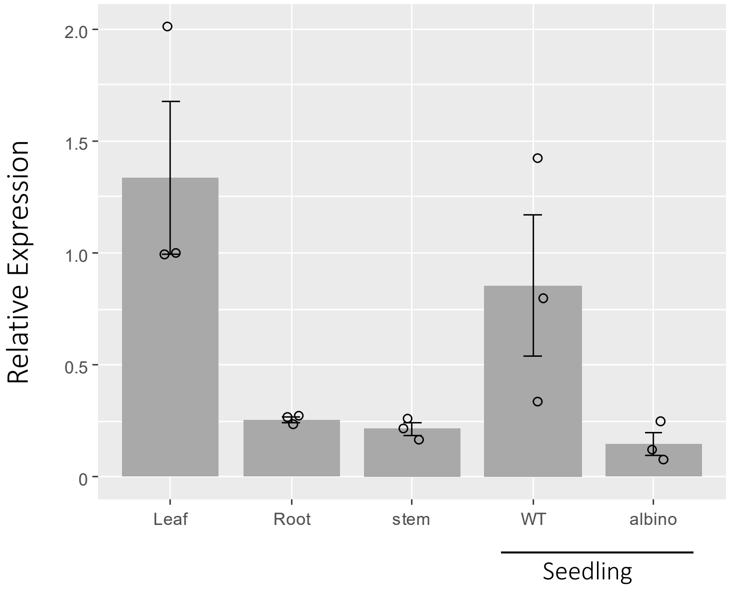

Supplementary Figure 12 | Expression of *CsMurE* in various tissues. Expression of *CsMurE* in wild-type (WT) and *alb1* was determined by qRT-PCR. *CsActin* was used as a reference. Data are presented as means ± standard errors (*n* = 3 biologycally independent samples).

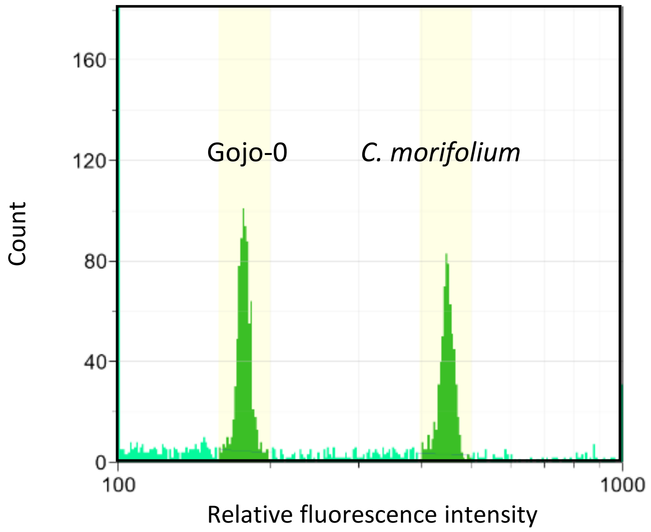

Supplementary Figure 13 | Estimation of genome size of *C. morifolium* by ploidy analyzer. Flow cytometric histograms of the nuclear suspension of *C. morifolium* var. 'Sei-marine' and Gojo-0. Genome size of 'Sei-marine' was estimated at 7.93 ± 0.04 Gb (n = 3) according to fluorescence intensity relative to Gojo-0.

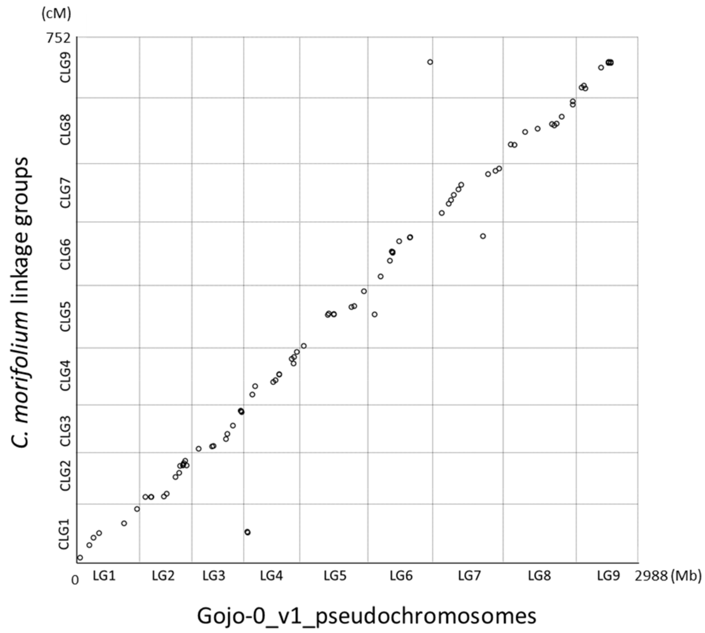

Supplementary Figure 14 | Comparison of Gojo-0 genomic sequences with genetic map of *C. morifolium.* The final assembly (Gojo-0_v1) was subjected to BLASTN search against 92 SNP markers of *C. morifolium*[1]. Markers showing significant hits (≤ 1e-10) were plotted on the pseudochromosomes of Gojo-0, and then linkage group names were assigned for individual chromosomes.

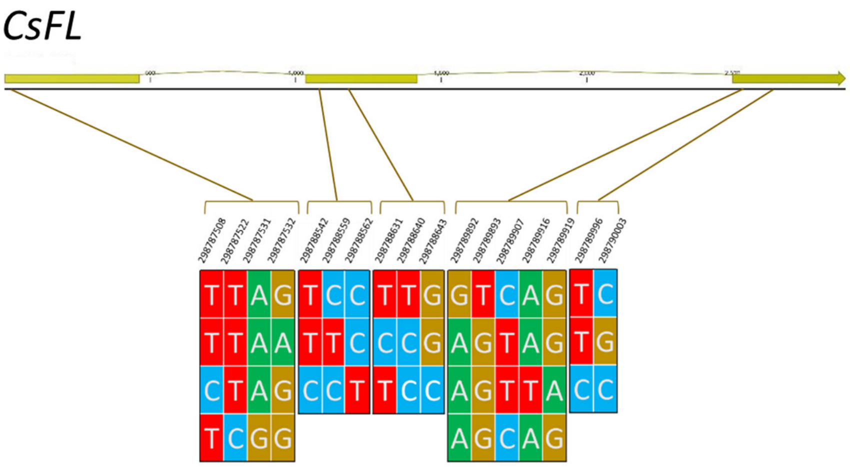

Supplementary Figure 15 | Haplotype structure of *CsFL* in cultivated chrysanthemum variety 'Jinbudiao'. Haplotype structures at five sites in *CsFL* based on RNA-seq data of the variety Jinbudiao are shown. The five haplotype blocks are independent.

Supplementary Table 1 | | Statistics of linkage groups.

Linkage group	Size(bp)	number of genes(HC)	%_repeatmasked
CsG_LG1	326,632,040	8,826	79.3
CsG_LG2	277,868,446	7,492	81.5
CsG_LG3	262,749,521	5,934	82.6
CsG_LG4	321,277,909	7,591	81.4
CsG_LG5	360,746,274	8,872	80.1
CsG_LG6	347,130,978	9,501	79.9
CsG_LG7	357,309,987	7,975	83.0
CsG_LG8	372,592,104	8,899	81.5
CsG_LG9	361,868,440	9,060	81.5
others	56,891,029	127	--
CsG_mitochondrion	208,120	--	--
CsG_chloroplast	150,960	--	--
Total	3,045,425,808	74,277	81.2

Supplementary Table 2 | Classification of intact LTR-RTs in the Gojo-0 genome.

Classification	Number
**Copia**	**18,820**
Ale	1,676
Angela	854
Bianca	21
Ivana	1,217
SIRE	5,125
TAR	1,315
Tork	267
Others	8,345
**Gypsy**	**15,085**
chromovirus	3,955
CRM	153
Galadriel	72
Reina	255
Tekay	2,109
non-chromovirus	10,251
Athila	8,512
Tat|Retand	1,732
Others	879
**unknown**	**23,446**
**Total**	**57,351**

Supplementary Table 3 | Classification of SbdRT copies.

SbdRT		Angela-type	
SbdRT-orf	161	150	
group1	46	45	autonomous
group2	15	14	autonomous
others	6	6	autonomous
fragmented	94	85	nonautonomous
SbdRT-nis	190	0	nonautonomous
SbdRT (others)	9	6	nonautonomous
Total	360	156	

SbdRT copies with long terminal repeats (LTRs) at both ends are shown. The total number of SbdRT was 360. Of the 161 SbdRT-orf copies, 67 were thought to be autonomous, and 150 were assigned to Angela-type LTR-RT.

Supplementary Table 4 | Accession numbers of raw data described in this study.

Platform		Library	Bioproject	Accessions	Description	Insert size	Total reads	Total bases
PacBio	Sequel	WGS	PRJDB7468	DRX233469	Single	13,744bp	24.9M	343Gb
Illumina	Hiseq2500	WGS	PRJDB7468	DRX142424	PE250	619bp	1,269M	317Gb
Illumina	HiseqX	Hi-C	PRJDB7468	DRX256944	PE150	--	1,586M	238Gb
PacBio	Sequel	Iso-seq	PRJDB5536	DRX274173	Single	--	--	--
				DRX274174	Single	--	--	--
				DRX274175	Single	--	--	--
				DRX274176	Single	--	--	--

Supplementary References

1. van Geest, G. et al. An ultra-dense integrated linkage map for hexaploid chrysanthemum enables multi-allelic QTL analysis. *Theor. Appl. Genet.* **130**, 2527–2541 (2017).

2. Oda, A. et al. *CsFTL3*, a chrysanthemum *FLOWERING LOCUS T-like* gene, is a key regulator of photoperiodic flowering in chrysanthemums. *J. Exp. Bot.* **63**, 1461–1477 (2012)

3. Shchennikova, A. V., Shulga, O. A., Immink, R., Skryabin, K. G. & Angenent, G. C. Identification and characterization of four chrysanthemum MADS-box genes, belonging to the Apetala1/Fruitfull and Sepallata3 subfamilies. *Plant Physiol.* **134**, 1632–1641 (2004)

4. Higuchi, Y. et al. The gated induction system of a systemic floral inhibitor, antiflorigen, determines obligate short-day flowering in chrysanthemums. *Proc. Natl. Acad. Sci. U. S. A.* **110**, 17137–17142 (2013).

5. Hirakawa, H. et al. De novo whole-genome assembly in Chrysanthemum seticuspe , a model species of chrysanthemums, and its application to genetic and gene discovery analysis. *DNA Res.* **26**, 195–203 (2019).

6. Ma, Y. P., Fang, X. H., Chen, F. & Dai, S. L. DFL, a FLORICAULA/LEAFY homologue gene from Dendranthema lavandulifolium is expressed both in the vegetative and reproductive tissues. *Plant Cell Rep.* **27**, 647–654 (2008).

7. Li, H. et al. The Sequence Alignment/Map format and SAMtools. *Bioinformatics* **25**, 2078–2079 (2009)

8. Huang, D. et al. Identification and characterization of *CYC*-like genes in regulation of ray floret development in *Chrysanthemum morifolium*. *Front. Plant Sci.* **7**, 1–15 (2016).
