# Supplementary material for: A chromosome-level genome sequence of Chrysanthemum seticuspe, a model species for hexaploid cultivated chrysanthemum
Source: Commun Biol. 2021 Oct 7;4:1167. doi: 10.1038/s42003-021-02704-y (PMC8497461; doi:10.1038/s42003-021-02704-y)
Supplement: Supplementary file 3 — Description of Additional Supplementary Files [file 42003_2021_2704_MOESM3_ESM.pdf]

Description of Additional Supplementary Files

File name: Supplementary Data 1

Description: **a -** The structures of SbdRTs in C. seticuspe and C. nankingense. a, DNA sequences of SbdRT-nis-type copies in C. seticuspe and C. nankingense are aligned. Yellow, pink, and blue boxes represent LTR, PBS-ATG, and NIS, respectively. Cs_LG2_264961294-264969213 and Cs_LG3_1449169269-1449177129 are SbdRTnis copies in C. seticuspe. Cn_utg2999_44585-52528 and Cn_utg57260_32948-40788 are SbdRT-nis copies in C. nankingense. Gray regions show sequence differences.

b - DNA sequences of SbdRT-orf-type copies in C. seticuspe and C. nankingense are aligned. Yellow, pink, and blue boxes indicate LTR, PBS-ATG, and ORF, respectively. Cs_LG5_64792825-64801000 and Cn_utg29575_47131-55244 are SbdRTorf in C. seticuspe and C. nankingense, respectively.

File name: Supplementary Data 2

Description: Sbd DNA sequences of SbdRTs in Supplementary Data 1.

File name: Supplementary Data 3

Description: SbdRTs identified in the Gojo-0 genome and their classification.

File name: Supplementary Data 4

Description: Floral organ genes predicted in the <i>C. seticuspe</i> genome (Gojo-0 v1).

File name: Supplementary Data 5

Description: SSR markers located on the Gojo-0 pseudochromosomes.

File name: Supplementary Data 6

Description: Homology of <i>C. seticuspe</i> genes compared with orthologs in cultivated chrysanthemum.

File name: Supplementary Data 7

Description: Primers used in this study.
