# Supplementary material for: A chromosome-level genome sequence of Chrysanthemum seticuspe, a model species for hexaploid cultivated chrysanthemum
Source: Commun Biol. 2021 Oct 7;4:1167. doi: 10.1038/s42003-021-02704-y (PMC8497461; doi:10.1038/s42003-021-02704-y)
Supplement: Supplementary file 4 — Supplementary data1 [file 42003_2021_2704_MOESM4_ESM.pdf]

a

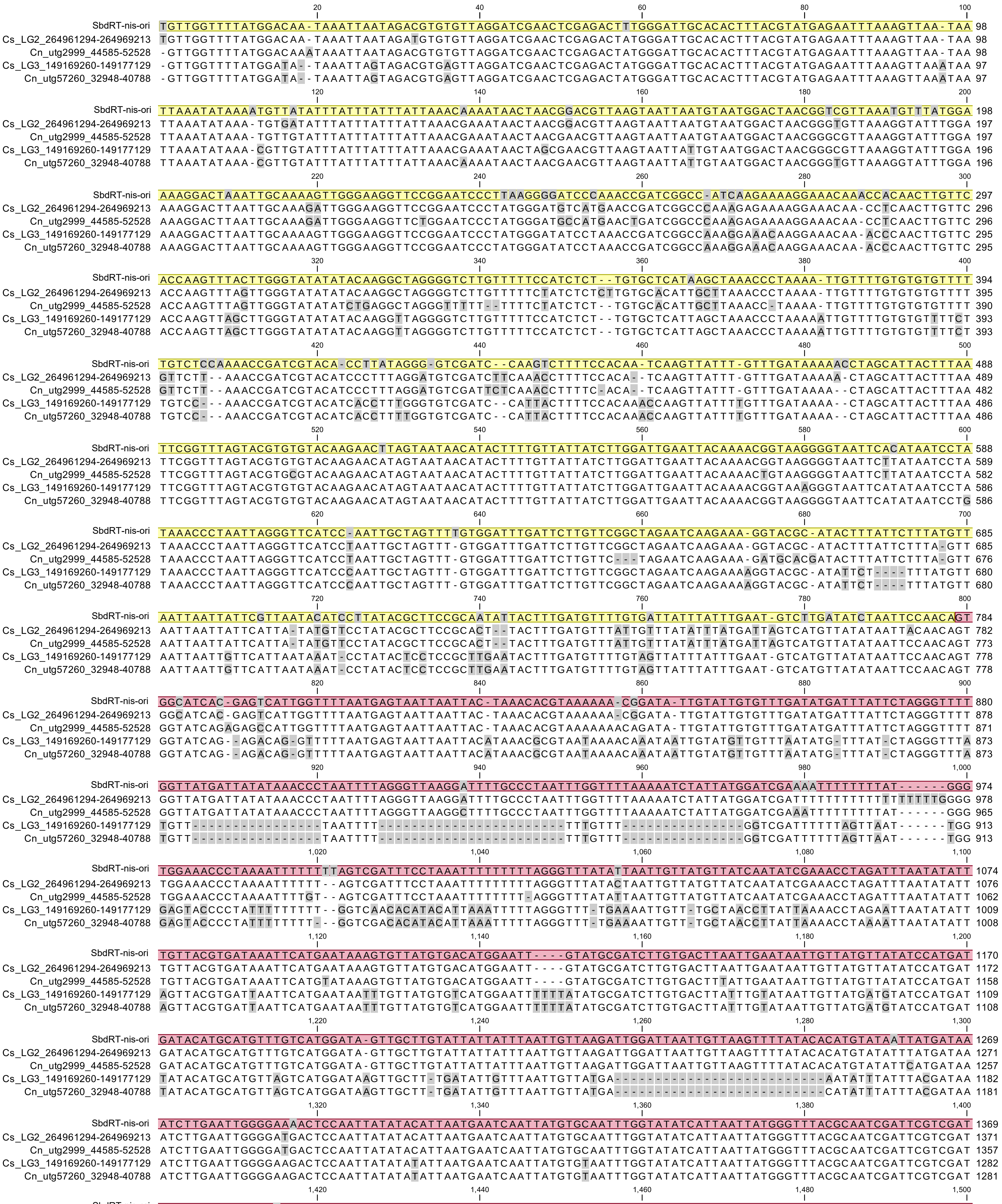
SbdRT-nis-ori
Cs_LG2_264961294-264969213
Cn_utg2999_44585-52528
Cs_LG3_149169260-149177129
Cn_utg57260_32948-40788

```
                          1,520               1,540               1,560               1,580               1,600
SbdRT-nis-ori             TGCATGTGGCTACTTGTTTACACGTATAAATTGTTTATTATGTGCGCATTGTTTTTAGTTGTTTATATTTTTTATTGGTATTACTTGTTTGTTTATTATG 1569
Cs_LG2_264961294-264969213 TGCATGTGGCTACTTGTTTACACGTATAAATTGTTTATTATGTGCGCATTGTTTTTAGTTGTTTATATTTTTTATTGGTATTACTTGTTTGTTTATTATG 1571
Cn_utg2999_44585-52528    TGCATGTGGCTACTTGTTTACACGTATAAATTGTTTATTATGTGTGCATTGTTTTTAGTTGTTTATATTTTT-ATTGGTATTACTTGTTTGTTTATTATG 1556
Cs_LG3_149169260-149177129 TGCATGTGGCTACTTGTTTACACGTATAAATTGTTTATTATGTGCGCATTGTTTTTTAGTAGTT-TATTTTTTATT--TGTTACTTGTTTGTTTATTATG 1479
Cn_utg57260_32948-40788   TGCATGTGGCTACTTGTTTACACGTATAAATTGTTTATTATA---GCATTGTTTTTTAGTAGTT-TATTTTTTATT--TGTTACTTGTTTGTTTATTATG 1475
                          1,620               1,640               1,660               1,680               1,700
SbdRT-nis-ori             TGCGACAG-TTTTAAGCAAAGGGTAATTTTGAATAGAAAAATAATACGGACTAAACAATGGCGAAATTTGATTTGTTTTGGAAGAGTTAAGTTTTCAATA 1668
Cs_LG2_264961294-264969213 TGCGACAG-TTTTAAGCAAAGGGTAATTTTGAATAGAAAAATAATACGGACTAAACAATGGTGAAATTTGATTTGTTTTGGAAGAGTTAAGTTTTCAATA 1670
Cn_utg2999_44585-52528    TGCGACAG-TTTTAAGCAAAGGGTAATTTTGAATAGAAAAATAATACGGACTAAACAATGGTGAAATTTGATTTGTTTTGGAAGAGTTAAGTTTTCAATA 1655
Cs_LG3_149169260-149177129 TTAGACAG-TTTTAAGCAAATGGTAATTTTGAATAGAAAAATAATACGGACTAAACAATGGCGAAATTTGATTTGTTTTGGAAGAGTTAAGTTTTCAATA 1578
Cn_utg57260_32948-40788   TTAGACAGGTTTTAAGCAAAGGGTAATTTTGAATAGAAAAATA-TACGGACTAAACAATGGCGAAATTTGATTTGTTTTGGAAGAGTTAAGTTTTCAATA 1574
                          1,720               1,740               1,760               1,780               1,800
SbdRT-nis-ori             GTTTAATTGATTGGTGACTTTGGTACAATGACTGAACGGCTCACCCATTCGAATGAACTATAAAGGGGTTATTTAAGAAGTGCGCACAGTCACACT-GAA 1767
Cs_LG2_264961294-264969213 GTTTAATTGATTGGTGACTTTGGTACAATGACTGAACGGCTCACCCATTCGAATGAACTATAAAGGGGTTATTTAAGAAGTGCGCACAGTCACACT-GAA 1769
Cn_utg2999_44585-52528    GTTTAATTGATTGGTGACTTTGGTACAATGACTGAACGGCTCACCCATTAGAATGAACTATAAAGGGGTTATTTAAGAAGTGC------TTACGCA-CAA 1748
Cs_LG3_149169260-149177129 GTTTAATTGATTGGTGACTTTGGTACAATGACTGAACGGCTCACCCATTCGAATGAACTATAAAGGGGTTATTTAAGAAGTGCGCACAGTCACACT-GAA 1677
Cn_utg57260_32948-40788   GTTTAATTGATTGGTGACTTTGGTACAATGG-TGAACGGCTCACCCATT-GAATGAACTATAAAGGG-TTATTTAAGAAGTGCGC-----CACACTTGAA 1666
                          1,820               1,840               1,860               1,880               1,900
SbdRT-nis-ori             AGGATGTCTAAGGTTATGCTTTTGCGCTTACCTGGTCCTCTGCCCTCTGTAAGTTCGAATGGAGCTACTTAGTAAGCCATAAAAGTACAAGCTCATCTTA 1867
Cs_LG2_264961294-264969213 TGGATGTCTAAGGTTATGCGTTTGCGCTTACCTGGTCCTCTGCCCTCTGTAAGTTCGAATGGAGCTACTTAGTAAGCCATAAAAGTACAAGCTCATCTTA 1869
Cn_utg2999_44585-52528    TGGATGTCTAAGGTTATGCGTTTGCGCTTACCTGGTCCTCTGCCCTCTGTAAGTTCGAATGGAGCTACTTAGTAAGCCATAAAAGTACAAGCTCATCTTA 1848
Cs_LG3_149169260-149177129 TGGATGTCTAAGGTTATGCGTTTGCGCTTACCTGGTCCTATGCCCTCTGTAAGTTCGAATGGAGCTACTTAGTAAGCCATAAAAGTACAAGCTCATCTTA 1777
Cn_utg57260_32948-40788   TGGATGTCTAAGGTTATGCTTT---GCTTACACGGTCCTATGCCCTC--TAAGTTCGAATGGAGCTACTTAGTAAGCCATAAAAGTACAAGCTCATCTTA 1761
                          1,920               1,940               1,960               1,980               2,000
SbdRT-nis-ori             AGGGCGAATTAGTTTTAATTCCATGCGCCACTTCTGTGGTAATTATCCGAAACACTAAGTGTTTACACTTATGCAAAAGTACGGTATAATGGACGATAAT 1967
Cs_LG2_264961294-264969213 AGGGCGAATTAGTTTTAATTCCATGCGCCACTTCGGTGGTAATTATCCGAAACACTAAGTGTTTACACTTATGCAAAAGTACGGTATAATGGATGATGAT 1969
Cn_utg2999_44585-52528    AGGGCGAATTAGTTTTAATTCCATGCGCCACTTCGGTGGTAATTATCCGAAACACTAAGTGTTTACACTTATGCAAAAGTACGGTATAATGGATGATAAT 1948
Cs_LG3_149169260-149177129 AGGGCGAATTAGTTTTAATTCCATGCGCCACTTCGGTGGTAATTATCCGAAACACTAAGTGTTTACACTTACGCAAAAGTACGGTATAATGGATGATAAT 1877
Cn_utg57260_32948-40788   AGG-CGAATTAGTTTTAATTCCAT-CGCCACTTCGGTGGTAATTATCC-AAACACTAAGTGTTTACACTTATGCAAAAGTGCGGTATAATGGATGATAAT 1858
                          2,020               2,040               2,060               2,080               2,100
SbdRT-nis-ori             TAACACTAAATTCCTTGATTGGTGAACCCAATACCTCAATCAAAACTTTGATAATTTGATCTTTATGAATTGGTAATAAAAAGTTTTTTATTAAAATTA 2067
Cs_LG2_264961294-264969213 TAACACTAAATTCCTTGATTGGTGAACCCAATACCTCAATCAAAACTTTGATAATTTGATCTTTATGAATTGGTAATAAAAAGTTTTT-ATTAAAATTA 2068
Cn_utg2999_44585-52528    TAACACTAAATTCCTTAATTGGTGAACCCAATACCTCAATCAAAACTTTGATAATTTGATCTTTATGAATTGGTAATAAAAAGGTTTTT-ATTAAAATTA 2047
Cs_LG3_149169260-149177129 TAACACTAAATTCCTTGATTGGTGAACCCAATACCTCAATCAAAACTTTGATAATTTGATCTTTATGAATTGGTAATAAAAAGTTTTT-ATTAAAATTA 1976
Cn_utg57260_32948-40788   TAACACTAAATTCCTTGATTGGTGAACCCAATACCTCAATCAAAACTTTGATAATTTGATCTTTATGAATTGGTAATAAAA-GTTTTT-ATTAAAATTA 1956
                          2,120               2,140               2,160               2,180               2,200
SbdRT-nis-ori             TGCTACAAGATGAATGCATGACATCTTTGTACATCTTTGATTTGACCACATAAAATCAAAACTAGTACCCTTTTAAATTAAG-AATATGCCATCTAATTA 2166
Cs_LG2_264961294-264969213 TGCTACAAGATGAATGCATGACATCTTTGTACATATTTGATTTGACCACATAAAATCAAAACTAGTACCCTTTTAAATTAAGGTATATGCCATCTAATTA 2168
Cn_utg2999_44585-52528    TGCTACAAGATGAATGCATGACATCTTTGTACATCTTTGATTTGACCACATAAAATCAAAACTAATACCCTTTTAAATTAAG-TATATGCCATCTAATTA 2146
Cs_LG3_149169260-149177129 TGCTACAAGATGAATGCATGACATCTTTGTACAAATTTGATTTGACCACATAAAATCAAAACTAGTACCCTTTTAAATTAAG-AATATGCCATCTAATTA 2075
Cn_utg57260_32948-40788   TGCTACAAGATGAATGCATGACATCTTTGTACAAATTTGATTTGACCACATAAAATCAAAACTAGTACCCTTTTAAATTAAG-AATATGCCATCTAATTA 2055
                          2,220               2,240               2,260               2,280               2,300
SbdRT-nis-ori             AAAGGAACACTTGAAGTTGCAATTTACGTCATGACAGTACTTGGCGAGATTGTAATGGAGAGGTAAATCTCTATGTAAAGTCAGACCTTACATGGACATA 2266
Cs_LG2_264961294-264969213 AAAGGAACACTTGAAGTTGCAATTTAAGTCATGAGAGTACTTGGCTAGATTGTGATGGAGAGGTAAATCTCTATGTTAAGTGAGACCTTACATGGACATA 2268
Cn_utg2999_44585-52528    AAAGGAACACTTGAAGTTGCAATTTAAGTCATGAGAGTACTTGGCTAGATTGTGATGGAGAGGTAAATCTCTATGTTAAGTGAGACCTTACATGGACATA 2246
Cs_LG3_149169260-149177129 AAAGGAACACTTGAAGTTGCAATTTACGTCATGACAGTACTTGGCTAGATTGTGATGGAGAGGTAAATCTCTATGTAAAGTCAGACCTTACATGGACATA 2175
Cn_utg57260_32948-40788   AA-GGAACACTTGAAGTTGCAATTTACGTCATGAC-----TTGGCTAGATTGTGATGGAGAGGTAAATCTCTATGTAAAGTC-GACCTTACATGGACATA 2148
                          2,320               2,340               2,360               2,380               2,400
SbdRT-nis-ori             TTTGCAGTTCTTTAAGTTAGACACTTAAGTTGAGTAGGAAGTGAGA-CCTGGACTCAATGGCATAAGATGGGATGAAACATGTTTATATTTGCAATAGCG 2365
Cs_LG2_264961294-264969213 TTTGCAGTTCTTTAAGTTAGACACTTAAGTTGAGTAGGAAGTGAGATCTTGGACTCAATGGCATAAGATGGGATGAAACATGTTTATATTTGCAATAGCG 2368
Cn_utg2999_44585-52528    TTTGCAGTTCTTTAAGTTAGACACTTAAGTTGAGTAGGAAGTGAGATCTTGGACTCAATGGCATAAGATGGGATGAAACATGTTTATATTTGCAATAGCG 2346
Cs_LG3_149169260-149177129 TTTGCAGTTCTTTAAGTTAGACACTTAAATTGAGTAGGAAGTGAGA-CCTGGACTCAATGGCATAAGATGGGATGAAACATGTTTATATTTGCAATAGCG 2274
Cn_utg57260_32948-40788   TTTGCGGTTCTTTAAGTTAGACACTTAAATTGAGTAGGAAGTGAGA-CACGGACTCAATGGCATAAGATGGGATGAAACATGTTTATATTTGCAATAGCG 2247
                          2,420               2,440               2,460               2,480               2,500
SbdRT-nis-ori             TTGGAAAATATAAATCCCATAATGATCTAGGAGTTGGAAATTGTTTGCCAATTAATGTTCGACAGATTACCTTAT-TATATGCGTTCGTTGCTAGC-AGA 2463
Cs_LG2_264961294-264969213 TTGGAAAATATAAATCCCATAATGATCTAGGAATTGGAAATTGTTTGCCAATTATTGTTCGACGGATTACCCTAT-TATGTGCTTTCGTTACTAGC-AGA 2466
Cn_utg2999_44585-52528    TTGGAAAATATAAATCCCATAATGATCTAGGAATTGGAAATTGTTTGCCAATTAATGTTCGACAGATTACCTTAT-TATGTGCTTTCGTTACTAGC-AGA 2444
Cs_LG3_149169260-149177129 TTGGAAAATATAAATCCCATAATGATCTAGGAGTTGGAAATTGTTTGCCAATTAGTGTTCGACAGATTACCCTAT-TATGTGCTTTCGTTACTAGC-AGA 2372
Cn_utg57260_32948-40788   TTGGAAAATATAAATCCCATAATGATCTAGGAGTTGGAAATTGTTTGCCAATTAGTGTTCGACAGATTACCTATTATATGTGCTTTCGTTACTAGCGAGA 2347
                          2,520               2,540               2,560               2,580               2,600
SbdRT-nis-ori             TCAACTGATGTAATGAACCTATGATAATTTGGATCCTAGTTTTCGAGAAAATACTATGGTTATATGTATATTACCTTGGGTATTTTATTTTCTTGAAAAG 2563
Cs_LG2_264961294-264969213 TCAACTGATGTAATGGAACTGCGATAATTTGGATCCTAGTTTTCGAGAAAATTCTATGGTTATATGAATATTACCTTGGGTATTTTATTTTCTTGAAAAG 2566
Cn_utg2999_44585-52528    TCAACTGATGTAATGGAACTATGATAATTTGGATCCTAGTTTTCGAGAAAATTCTATGGTTATATGAATATTACCTTGGGTATTTTATTTTCTTGAAAAG 2544
Cs_LG3_149169260-149177129 TCAACTGATGTAATGGAACTACGATAATTTGGATCCTAGTTTTCGAGAAAATTCTATGGTTATATGAATATTACCTTGGGTATTTTATTTTCATGAAAAG 2472
Cn_utg57260_32948-40788   TCAAC-GATGTAATGGAACTATGATAATTTGGATCCTAGTTTTCGAGAAAATTCTATGGTTATATGAATATTACCTTGGGTATTTTATTTTCTTGAAAAG 2446
                          2,620               2,640               2,660               2,680               2,700
SbdRT-nis-ori             TTTAATTATCGAAAATACTAAAATTGAATTTTGCTAATTCTTTAAATGTATTAATGCAACATTAAACCGTAAACGAAGGTTATTCCTGTATTGGTAAA-T 2662
Cs_LG2_264961294-264969213 TTTAATTATCGAAAATACTAAAATTGAATTTTGCTAATTCTTTAAATGTATTAATGCAACATTAAACCGTAAACGTAGGTTATTCATGTATTGGTAAAAT 2666
Cn_utg2999_44585-52528    TTTAATTATCGAAAATACTAAAATTGAATTTTGCTAATTCTTTAAATGTATTAATGCAACATTAAATCGTAAACGTAGGTTATTCTTGTATTGGTAAA-T 2643
Cs_LG3_149169260-149177129 TTTAATTATCGAAAATACTAAAATTGAATTTTGCTAATTCTTTAAATGTATTAATGCAACATTAAATCGTAAACGTAGGTTATTCTTGTATTGGTAAA-T 2571
Cn_utg57260_32948-40788   TTTAATTATCGAAAATACTAAAATTGAATTTTGCTAATTCTTTAAATGTATTAATGCAACATTAAATCGTAAACGTAGGTTATTCTTGTATTGGTAAA-T 2545
                          2,720               2,740               2,760               2,780               2,800
SbdRT-nis-ori             TGTTCTACACCTGTAAGGGTTTT-TGGTAGAGTCAACTTTACTAAAAGAATAAACCTAGTCCTCTTTTC-TAAGAAAGAGTGAGATTCAATCCTATTCCT 2760
Cs_LG2_264961294-264969213 TGATCTACACCTGTAAGGGTTT--TGGTAGAGTCAACTTTACTAAAAGAATAAACCTAGTCCTCTTTTC-TAAGAAAGAGTGAGATTCAACCCTATTCCT 2763
Cn_utg2999_44585-52528    TGATCTACACCTGTAAGGGTTTTTTGGTAGAGTCAACTTTACTAAGAGAATAAACCTAGTCCTCTTTTCATAAGAAAGAGTGAGATTCAACCCTATTCCT 2743
Cs_LG3_149169260-149177129 TGATCTACACCTGTAAGGGTTTT-TGGTAGAGTCAAATTTACTAAAAGAATAAACCTAGTCCTCTTTTCACGCGAAAGAGTGAGATTCAACCCTATTCCT 2670
Cn_utg57260_32948-40788   TGATCTACACCTGTAAGGGTTTT-TGGTAGAGTCAACTTTACTAAAAGAATAAACCTAGTCCTCTTTTCACGCGAAAGAGTGAGATTCAACCCTATTCCT 2644
                          2,820               2,840               2,860               2,880               2,900
SbdRT-nis-ori             TTTCATGAATGAAGAAAGGTATGATAGGACAATTAGAATTGTCTAAATTCTAAATACATAACTCGTAAGCAAATTTTAATAGTTAAGAAAATTGCATATC 2860
Cs_LG2_264961294-264969213 TTTCATGAATGAAGAAAGGTATGATAGGACAATTAGAATTGTCTAAATTCTAAATACATAACTCGTAAGCAAATTTTAATAGTTAAGAAAATTGCATATT 2863
Cn_utg2999_44585-52528    TTTCATGAATGAAGAAAGGTATGATAGGACAATTAGAATTGTCTAAATTCTAAATACATAACTCGTAAGCAAATTTTAATAGTTAAGAAAATTGCATATT 2843
Cs_LG3_149169260-149177129 TTTCATGAATGAAGAAAGGTATGATAGGACAATTAGAATTGTCTAAATTCTAGATACATAACTCGTAAGCGAATTTTAATAGTTAAGAAAATTGCATATT 2770
Cn_utg57260_32948-40788   TTTCATGAATGAAGAAAGGTATGATAGGACAATTAGAATTGTCTAAATTCTAGATACATAACTCGTAAGCGAATTTTAATAGTTAAGAAAATTGCATATT 2744
                          2,920               2,940               2,960               2,980               3,000
SbdRT-nis-ori             TCTGAGTTATTAAATTAGAAGCTAGACTAATTACTGTATAATTGGTAAAAGACAAACTAGCATATTTTATGTAGTTGTT-TGTTTTGACCATTTAAAAGT 2959
Cs_LG2_264961294-264969213 TCTGAGTTATTAAATTAGAAGCTAGACTAATTACTGTATAATTGGTAAAAGACAAACTAACATATTTTATCTAGTTGTTGTGCTTTGACCATTTAAAAGT 2963
Cn_utg2999_44585-52528    TCTGAGTTATTAAATTAGAAGCTAGACTAATTACTGTATAATTGGTAAAAGACAAACTAGCATATTTTATCTAGTTGTTTTGTTTTGACCATTTAAAAGT 2943
Cs_LG3_149169260-149177129 TCTGAGTTATTAAATTAGAAGCTAGACTAATTACTGTATAATTGGTAAAAGACAAACTAGCATATTTTATCTAGTTGTTGCACTTTGACCATTTAAAAGT 2870
Cn_utg57260_32948-40788   TTTGAGTTATTAAATTAGAAGCTAGACTAATTACTGTATAATTGGTAAAAGACAAACTAGCATATTTTATCTAGTTGTTGTACTTTGACCATTTAAAAGT 2844
```

```
                                       3,020       3,040       3,060       3,080       3,100
SbdRT-nis-ori                ACTACTTACAATTGTTGGAATAGTAGAACTATTAAGAAAGAAATTGCATGCCCAATTTTTCAAGTTAATAGCATTGATGAATAA-TTCATCATCTACATA 3058
Cs_LG2_264961294-264969213   ACTACTTACAATTGTTGGAATAGTAGAACTATTAAGAAAGAAATTGCATGCCCAATTTTTCAAGTTAATAGCATTGATGGATAG-TTCATCATCTACATA 3062
Cn_utg2999_44585-52528       ACTACTTACAATCGTTGGAATAGTAGAACTATTAAGAAAGAAATTGCATGCCCAAATTTTCAAGTTAATAGCATTGATGGATAAATTCATCATCTACATA 3043
Cs_LG3_149169260-149177129   ACTACTTACAATTGTTGGAATAGTATAACTATTAAGAAAGAAATTACATGCCCAAATTTTCAAGTTAATAGCATTGATGGATAAATTCATCATCTACATA 2970
Cn_utg57260_32948-40788      ACTACTTACAATTGTTGGAATAGTAGAACTATTAAGAAAGAAATTACATGCCCAATTTTTCAAGTTAATAGCATTGATGGATAAATTCATCATCTACATA 2944

                                       3,120       3,140       3,160       3,180       3,200
SbdRT-nis-ori                GTAGAATACATTTTTTTAAGTACATAAGTAATTGTTATGTATGGACTTTCTCAAAGGTGTAGG-AAGTAAATAATACACTATTTTCTTTCACTGGTTAAT 3157
Cs_LG2_264961294-264969213   GTAGAATACACTCTTT-AAGTACATAAGTAATTGTTATGTATGGACTTTTTCAAAGGTGTAGGGAAGTAAATAGAACACTATTTTCTTTTACTGGTTAAT 3161
Cn_utg2999_44585-52528       GTAGAATACATTCTTTTAAGTACATAAGTAATTGTTATGTATGGACTTTCTCAAAGGTGTAGGGAAGTAAATAGAACACTATTTTCTTTTACTGGTTAAT 3143
Cs_LG3_149169260-149177129   GTAGAATACATTCTTTTAAGTACATTTGTAATTGTTATGTATGGACTTTCTCAAAGGTGTAGG-AAGTAAATAATACACTATTTTCTTTCACTGGTTAAT 3069
Cn_utg57260_32948-40788      GTAGAATACATTCTTTTAAGTACATTTGTAATTGTTATGTATGTACTTTCTCAAAGGTGTAGG-AAGTAAATAATACACTATTTTCTTTCACTGGTTAAT 3043

                                       3,220       3,240       3,260       3,280       3,300
SbdRT-nis-ori                GAAACACTTACAATGTTGTCAAATCCAGTGGGAGTGTTGTGTTCTTATTACTGAATAAGGAATTAATGTAATGATCTCTTATTCAGTGGGAGTTACAAGC 3257
Cs_LG2_264961294-264969213   GAAACACTTCCAATGTTGTCAAATCCAGTGGGAGTATGTTGTTCTTATTACTGAATAAGGACTTAATGTAATGATCTCTTATTCAGTGGGAGTTACAAGC 3261
Cn_utg2999_44585-52528       GCAACACTTACAATGTTGTCAAATCCAGTGGGAGTGTTGTGTTCTTATTACTGAATAAGGAATTAATGTAATGATCTCTTATTCAGTGGGAGTTACAAGC 3243
Cs_LG3_149169260-149177129   GAAACACTTACAATGTTGTCAAATCCAGTGGGAGTGTTGTGTTCTTATTACTGAATAAGGAATTAATGTAATGATCTCTTATTTAGTGGGAGTTACAAGC 3169
Cn_utg57260_32948-40788      GAAACACTTACAATGTTGTCAAATCCAGTGGGAGTGTTGTGTTCTTATTACTGAATAAGGAATTAATGTAATTATCTCTTATTCAGTGGGAGTTACAAGC 3143

                                       3,320       3,340       3,360       3,380       3,400
SbdRT-nis-ori                TTTGTCATATTGGTAGTAACCCAGATTATCTCTAGAATGTCTTAAGATAATTACTTTCTACTTGTAGACACTTATGTTTTCAAGGATAGTCATGTACGTT 3357
Cs_LG2_264961294-264969213   TTTGTCATATTGGTAGTAACTCAGATTATCTCTAGAATGTCTTATGATAATTACTTTCCACTTGTAGACACTTATGTTTTCAAGGATAGTCATGTACGTT 3361
Cn_utg2999_44585-52528       TTTGTCATCTTGGTAGTAACCCAGATTATCTCTAGAATGTCTTAAGATAATTACTTTCCACTTGAAGACACTTATGTTTTCAAGGATAGTCATGTACGTT 3343
Cs_LG3_149169260-149177129   TTTGTCATCTTGGTAGTAACCCATATTATCTCTTGAATGTCTTAAGATAATTACTTTCCACTTATAGACACTTATGTTTTCAAGGATAGTCATGTACGTT 3269
Cn_utg57260_32948-40788      TTTGTCATCTTGGTAGTAACCCAGATTATCTCTAGAATGTCTTAAGATAATTACTTTCCACTTATAGACACTTATGTTTTCAAGGATAGTCATGCACGTT 3243

                                       3,420       3,440       3,460       3,480       3,500
SbdRT-nis-ori                TCCTTATTAGACAATAAAGTGGTTCTACATCTACTAGTTATGCTAATATCAATGACACAATCATATTAGTCACATAAACAAACATATGTCGTGTTGTTTA 3457
Cs_LG2_264961294-264969213   TCCTTATTAGACAATGAAGTGATTCTACATCTACTAGTTATGCTAATATCAATGACACAATCATATTAGTCACATAAACAAACATATGTCGTGTTGTTTA 3461
Cn_utg2999_44585-52528       TCCTTATTAGACAATGAAGTGGTTCTACATCTACTAGTTATGCTAATATCAATGACACAATCATATTAGTCACATAAACAAACATATGTCGTGTTGTTTA 3443
Cs_LG3_149169260-149177129   TCCTTATTAGACAATGAAGTGGTTCTACATCTACTAGTTATGCTAATATCAATGACACAATCATATTAGTCACATAAACAAACATATGTCGTGTTGTTTA 3369
Cn_utg57260_32948-40788      TCCTTATTAGACAATGAAGTGGTTCTACATCTACTAGTTATGCTAATATCAATGACACAATCATATTAGTCACATAAACAAACATATGTCGTGTTGTTTA 3343

                                       3,520       3,540       3,560       3,580       3,600
SbdRT-nis-ori                TTTGCTACATGATGTTGTTCCATGCACAAGTGGGAGAAATCTATATTGTGTGCAAGGTACAACATATAGTTATATGATTTAATAGATTCTCTTGACTGCA 3557
Cs_LG2_264961294-264969213   TCTGCTACATGATGTTGTTCCATGCACAAGTGGGAGAAATTTATATTGTGTGCAAGGTACAACATATAAGTATATGATTTAAAGGATTCTCTTGACTGCA 3561
Cn_utg2999_44585-52528       TCTGCTACATGATGTTGTTACATGCACAAGTGGGAGAAATCTATATTGTGTGCAAGGTACAACATATAAGTATATGATTTAACGGATTCTCTTGACTGCA 3543
Cs_LG3_149169260-149177129   TCTGCTACATGATTTTGTTTCATGCACAAGTGGGAGAAATCTATATTGTGTGCAAGGGACAACATATAGGTATATGATTTAAAGGATTCTCTTGACTGCA 3469
Cn_utg57260_32948-40788      TCTGCTACATGATTTTGTTCCATGCACAAGTGGGAGAAATCTATATTGTGTGCAAGGGACAACATATAGGTATATGATTTAAAGGATTCTCTTGACTGCA 3443

                                       3,620       3,640       3,660       3,680       3,700
SbdRT-nis-ori                AGGTAAGAGACTAACATTCTAATATAACTTGTACATATCTAATTCAGGAAACATTTGGTGTTGCAATTTACGTCATGTCAGAACTTGGCGAGATTGCAAT 3657
Cs_LG2_264961294-264969213   AGGTAAGAGACTAACGTTCTAATATAACTTGTACATATCTAATTCAAGAAACACTTGGTGTTACAATTTACGTCATGTGAGAACTTGGCGAGATTGCAAC 3661
Cn_utg2999_44585-52528       AGGTAAGAGACTAACGTTCTAATATAACTTGTACATATCTAATTCAAGAAACACTTGGTGTTACAATTTACGTCATGTGAGAACTTGGCGAGATTGCAAC 3643
Cs_LG3_149169260-149177129   AGGTAAGAAACTAACGTTCTAATATAAATTGTACATATCTAATTCAAGAAACACTTGGTGTTGCAATTTACGTCATGTCAGAACTTGGCGAGATTGCAGC 3569
Cn_utg57260_32948-40788      AGGTAAGAAACTAACGTTCTAATATAACTTGTACATATCTAATTCAAGAACCACTTGGTGTTGCAATTTACGTCATGTCAGAACTTGGCGAGATTGCAGC 3543

                                       3,720       3,740       3,760       3,780       3,800
SbdRT-nis-ori                AACAAAGTAAATCTCTATGTGAAGTCAGACCTTGCATGGACATATTTACAGTTTCTAAATTAGACAAGTATTTAGAACATGTGTCAAAATGATATATAAG 3757
Cs_LG2_264961294-264969213   AACAAAGTAAATCTCTATGTGAAGTCAGACCTTGCATGGACATATTTGCAGTTTCTAAATTAGACAAGTATTTAGAACATGTGTCAAAATGATATATAAG 3761
Cn_utg2999_44585-52528       AACAAAGTAAATCTCTATGTAAAGTGAGATCTTGCATGGACATATTTACAGTTTCTAAATTAGACAAGTATTTAGAACATGTGTCAAAATGATATATAAG 3743
Cs_LG3_149169260-149177129   AACAAAGTAAGTCTCTATGTGAAGTCAGACCTTGCATGGACATATTTGCAGTTTCTAAATTAGACAAGTATTTAGAACATGTGTCAAAATGATATATAAG 3669
Cn_utg57260_32948-40788      AACAAAGTAAGTCTCTATGTGAAGTCAGACCTTGCATGGACATATTTGCAGTTTCTAAATTAGACAAGTATTTAGAACATGTGTCAAAATGATATATAAG 3643

                                       3,820       3,840       3,860       3,880       3,900
SbdRT-nis-ori                ACTACTTCAAATTAAATATTTATGTATTATTAGAATGACATAAAATTAAATAAAGTAATATAATCCTACTTCAA-TTTGTTTATTTATGTATTTTGGAAT 3856
Cs_LG2_264961294-264969213   ACTACTTCAAATTAGATTTTTATGTATTATTAGAATGACATAAA------TGAAGTAATCTAATACTACTTCAAATTTGTTTATTTATGTATTTTGGAAT 3855
Cn_utg2999_44585-52528       ACTACTTCAAATTAGATTTTTATGTATTATTAGAATGACATAAA------TGAAGTAATCTAATACTATTTCAAATTTGTTTATTTATGTATTTTGGAAT 3837
Cs_LG3_149169260-149177129   ACTACTTCAAATTAAATATTTATGTATTATCAGAATGACATAAAATTAAATAAAGTAATCTAATCCTACTTCAA-TTTGTTTATTTATGTATTTTGGAAT 3768
Cn_utg57260_32948-40788      ACTACTTCAAATTAAATATTTATGTATTATTAGAATGACATAAAATTAAATAAAGTAATCTAATCCTACTTCAA-TTTGTTTATTTATGTATTTTGGAAT 3742

                                       3,920       3,940       3,960       3,980       4,000
SbdRT-nis-ori                GACATAAATGAAAAATAATGTAGTCCTACATTATTATGAAGTAAAATAAATTAACTTGAAATTACTATTGAAACCAAGAGTTGTGTTCATAATTACTTTA 3956
Cs_LG2_264961294-264969213   GACATAAATGAAAAATAATGTAGTCCTGCATTATTGTGAAGTAAAATAAATTAACTTGAAATTACTATTGAAACCAAGAGTTGTGTTCATAATTACTTTA 3955
Cn_utg2999_44585-52528       GACATAAATGAAAAATAATGTAGTCCTACATTATTGTGAAGTAAAATAAATTAACTTGAAATTACTATTGAAACCAAGAGTTGTGTTCATAATTACTTTA 3937
Cs_LG3_149169260-149177129   GACATAAATGAAAAATAATGTAGTCCTACATTATTATGAAGTAAAATAAATTAACTTGAAATTACTATTGAAACCAAGAGTTGTGTTCATAATTACTTTA 3868
Cn_utg57260_32948-40788      GACATAAATGAAAAATAATGTAGTCCTACATTATTATGAAGTAAAATAAATTAACTTGAAATTACTATTGAAACCAAGAGTTGTGTTCATAATTACTTTA 3842

                                       4,020       4,040       4,060       4,080       4,100
SbdRT-nis-ori                GTTAAACATGTTTATGATTCATTGTAAAGACATAAACATAAAGTCTTTTAAGACTTATAATAAAGTAAGACGTTAACTTGTTATGTTTTTT-AATTAAGA 4055
Cs_LG2_264961294-264969213   GTTAAACATGTTTATGATTCATTGTAAAGACATAAACATAAAGTCTTTTAAGACTTATAATAAAGTAAGACGTTAACTTGTTATGTTTTTT-AATTAAGA 4054
Cn_utg2999_44585-52528       GTTAAACATGTTTATGATTCATTGTAAAGACATAAACATAAAGTCTTT-AAGACTTATAATAAAGTAAGACGTTAACTTGTTATGTTTTTT-AATTAAGA 4035
Cs_LG3_149169260-149177129   GTTAAACATGTTTATGATTCATTGTAAATACATAAACATAAAGTCTTTTAAGACTTATAATAAAGTAAGACGTTAACTTGTTATGTTTTTT-AATTAAGA 3967
Cn_utg57260_32948-40788      GTTAAACATGTTTATGATTCATTGTAAAGACATAAACATAAAGTCTTTTAAGACTTATAATAAAGTAAGACGTTAACTTGTTATGTTTTTTTAATTAAGA 3942

                                       4,120       4,140       4,160       4,180       4,200
SbdRT-nis-ori                CATATACAAGAAGTCATTGAGTTGATCAATGATTTATAACGTATAATGGGATTATAACAGTTTATGTTTCTGGTTGAGACATGGATATGAAGTGACTTGG 4155
Cs_LG2_264961294-264969213   CATATACAAGAAGTCAT---------CAATGATTTATAACGTATAATGGGATTATAACAGTTTATGTTTCTTGTTGAGACATGGATATGAAGTGACTTGG 4145
Cn_utg2999_44585-52528       CATATACAAGAAGTCATTGAGTTGATCAATGATTTATAACGTATAATGGGATTATAACAGTTTATGTTTCTTGTTGAGACATGGATATGAAGTAACTTGG 4135
Cs_LG3_149169260-149177129   CATATACAAGAAGTCATTGAGTTGATCAATGATTTATAACGTATAATGGGATTATAACAGTTTATGTTTCTTTTTGAGACATGGATATGAAGTGACTTGG 4067
Cn_utg57260_32948-40788      CATATACAAGAAGTCATTGAGTTGATCAATGATTTATAACGTATAATGGGATTATAACAGTTTATGTTTCTTGTTGAGACATGGATATGAAGTGACTTGG 4042

                                       4,220       4,240       4,260       4,280       4,300
SbdRT-nis-ori                TTAAAAGGTACTTGTGTTATAAAGCATGCAATTGGCTTGGAAAGTCTATTATACCATTAAGTTGACTTAGTTGGTAAATAGGTAAGCCAAAAGGAATATG 4255
Cs_LG2_264961294-264969213   TTAAAAGGTACTTGTGTTATAAAGCATGCAATTGGCTTGGAAAGTCTATTATACCATTAAGTTGACTTAGTTGGTAAATAGGAAATTCAAAATGAATATG 4245
Cn_utg2999_44585-52528       TTAAAAGGTACTTGTGTTATAAAGCATGCAATTGGCTTGGAAAGTCTATTATACCATTAAGTTGACTTAGTTGGTAAATAGGTAATTCAAAATGAATATG 4235
Cs_LG3_149169260-149177129   TTAAAAGGTACTTGTGTTATAAAGCATGTAATTGGCTTGAAAAGTCTATTATACCATTAAGTTGACTTAGTTGGTAAATAGGTAAGCCAAAAGGAATATG 4167
Cn_utg57260_32948-40788      TTAAAAGGTACTTGTGTTATAAAGCATGTAATTGGCTTGAAAAGTCTATTATACCATTAAGTTGACTTAGTTGGTAAATAGGTAAGCCAAAAGGAATATG 4142

                                       4,320       4,340       4,360       4,380       4,400
SbdRT-nis-ori                TTTATCTTATATATTGTTAGATTGTCTTACAATATTAAA-TTCTAATGAATACCACAAGTTGATGGTATGTTCAGTTAAGAGAATCAAACTCCATTTGAT 4354
Cs_LG2_264961294-264969213   TTTATCTTATATATTGTTAGATTGTCTTACAATATTAAAATTCTAATGAATACCACAAGTTGATGGTATGTTCAGTTAGGAGAATCAAACTCTATTTGAT 4345
Cn_utg2999_44585-52528       TTTATCTTATATATTGTTAGATTGTCTTACAATATTAAA-TTCTAATGAATACCACAAGTTGATGGTATGGTCAGTTAGAAGAATCAAACTCTATTTGAT 4334
Cs_LG3_149169260-149177129   TTTATCTTATATATTGTTAGATTGTCTTACAATATTAAA-TTCTAATGAATACCACAAGTTGATGGTATGTTCAGTTAGGAGAATCAAACTCTATTTGAT 4266
Cn_utg57260_32948-40788      TTTATCTTATATATTGTTAGATTGTCTTACAATATTAAA-TTCTAATGAATACCACAAGTTGATGGTATGTTCAGTTAGGAGAATCAAACTCTATTTGAT 4241

                                       4,420       4,440       4,460       4,480       4,500
SbdRT-nis-ori                TTGTTTGATTCATGATAACCATAACTTCTTTCTTAAAAAGTAACAAGGTTAGAATATTGAGTATGGATTGAATATACTCAATATGACTCCAACCTAGAAG 4454
Cs_LG2_264961294-264969213   TTGTTTGATTCAAGATAACCATAACTTCTTTCTTAAAAAGTAACAAGGTTAGAATATTGAGTATGGATTGAATATACTCAATATGACTCCAACCTAGAAG 4445
Cn_utg2999_44585-52528       TTGTTTGATTCAAGATAACCATAACTTCTTTCTTAAAAAGTAACAAGGTTAGAAAATTGAGTATGGATTGAATATACTCAATATGACTCCAACCTAGAAG 4434
Cs_LG3_149169260-149177129   -----TGATTCAAGATAACCATAACTTCTTTCTTAAAAAGTAACAAGGTTAGAAAATTGAGTATGGATTAAATATACTCAATATGACTCCAACCTAGAAG 4361
Cn_utg57260_32948-40788      -----TGATTCAAGATAACCATAACTTCTTTCTTAAAAAGTAACAAGGTTAGAAAATTGAGTATGGATTAAATATACTCAATATGACTCCAACCTAGAAG 4336
```

```
                                  4,520               4,540               4,560               4,580               4,600
SbdRT-nis-ori              ATTAATAAAACA-CCATTTCATTATGGAATGTGATGAATCACATTAAGTTTTAAAGTTGAAGATTCTGAATTTGTTAAACTACTTATGAATAGGTTATTA 4553
Cs_LG2_264961294-264969213 ATTAATAAAACA-CCATTTCATTATGGAATGTGATGAATCACATTAAGTTTTAAAATTGAAGATTCTGAATTTGTTAAACTACTTATGAATAGGTTATTA 4544
Cn_utg2999_44585-52528     ATTAATAAAACA-CCATTTCATTATGGAATGTGATGAATCACATTAAGTTTTAAAATTGAAGATTCTGAATTTGTTAAACTACTTATGAATAGGTTATTA 4533
Cs_LG3_149169260-149177129 ATTGATAAAAAAACCATTTTATTATGGAATGTGATGAATCACATTAAGTTTTAAAATTGAAGATTCTGAATTTGTTAAACTACTTATGAATAGGTTATTA 4461
Cn_utg57260_32948-40788    ATTGATAAAACA-CCATTTTATTATGGAATGTGATGAATCACATTAAGTTTTAAAATTGAAGATTCTGAATTTGTTAAACTACTTATGAATAGGTTATTA 4435
                                  4,620               4,640               4,660               4,680               4,700
SbdRT-nis-ori              ACAAACTAATGTCAAGAATTATCAATATGTATTTGTAATGTCCCAAATAGAGAACAATGTAGTTTTGCTCTAATTGCTAAGTATATATAGATAGTCTATA 4653
Cs_LG2_264961294-264969213 ACAAACTAATGTCAAGAATTATCAATGTGTATTTGTAATGTCCCAAGTAGAGAACAATGTAGTTTTGCTCTAATTGCTAAGTATATATAGATAGTCTATA 4644
Cn_utg2999_44585-52528     ACAAACTTAAGTCAGGAATTATCAATGTGTATTTGTAATGTCCCAAATAGAGAACAATGTAGTTTTGCTCTAATTGCTAAGTATATATAGATAGTCTATA 4633
Cs_LG3_149169260-149177129 ACAACGTAAAGTCAGGAATTATCAATGTGTATTTGTAATGTCCCAAAAAGAGAACAATGTAGTTTTGCTCTAATTGCTAAGTATATATAGATAGTCTATA 4561
Cn_utg57260_32948-40788    ACAACGTAAAGTTAGGAATTATCAATGTGTATTTGTAATGTCCCAAAAAGAGAACAATGTAGTTTTGCTCTAATTGCTAAGTATATATAGATAGTCTATA 4535
                                  4,720               4,740               4,760               4,780               4,800
SbdRT-nis-ori              TAT--TACAAAAGGCAAGTGGGAGTTTTTTTATCTTGAAAGATAAAATTACAAGATACATAATCTTCTAAAACACACTAGTAAATATCATGATGAGGTTA 4751
Cs_LG2_264961294-264969213 TAT--TACAAAAGGCAAGTGGGAGTTTTTT-ATCTTGAAAGATAAAATTACAAGATACATAATCTTCTAAAACACACTAGTAAATATCATGATGAGGTTA 4741
Cn_utg2999_44585-52528     TAT--TACAAAAGGCAAGTGGGAGTTTTTT-ATCTTGAAAGATAAAATTACAAGATACATAATCTTCTAAAACACACTAGTAAATATCATGATGAGGTTA 4730
Cs_LG3_149169260-149177129 TATATTACAAAAGGCAAGTGGGAGTTTT---ATCTTGAAAGATAAAATTACAAGATACATAATCTTCTAAAACACACTAGTAAATATCATGATGAGGTTA 4658
Cn_utg57260_32948-40788    TATATTACAAAAGGCAAGTGGGAGTTTT---ATCTTGAAAGATAAAATTACAAGATACATAATCTTCTAAAACACACTAGTAAATATCATGATGAGGTTA 4632
                                  4,820               4,840               4,860               4,880               4,900
SbdRT-nis-ori              GACATGTAATTGATAGACCTCATGATGATGGAATTTTCATTCATAGATCTGCAAGGATATCTCAGATATTTGACTTATATGCTGAGAAATATGAGTTACA 4851
Cs_LG2_264961294-264969213 GACATGTAAATGATAGACCTCATGATGATGGAATTTTCATTCATAGATCTGCAAAGATATCTCAGATATTTGACTTATATGCTGAGAAATATGAGTTACA 4841
Cn_utg2999_44585-52528     GACATGTAAATGATAGACCTCATGATGATGGAATTTTCATTCATAGATCTGCAAGGATATCTCATATATTTGATTTATATGCTGAGAAATATGAGTTACA 4830
Cs_LG3_149169260-149177129 GACATGTAAATGATAGACCTCATGATGATGGAATTTTCATTCATAAATCTGCAAGGATATCTCAGATATTTGACTTATATGCTGAGAAATATGAGTTACA 4758
Cn_utg57260_32948-40788    GACATGTAAATGATAGACCTCATGATGATGGAATTTTTATTCATAGATCTGCAAGGATATCTCAGATATTTGACTTATATGCTGAGAAATATGAGTTACA 4732
                                  4,920               4,940               4,960               4,980               5,000
SbdRT-nis-ori              GGATTATAGTAAACCTGATAATCATTATGATGGATTACCAAATATTGAATTGGGTAAATTACTCATACTATGAATGTTAAAGATGCTGTCCATAAAGATA 4951
Cs_LG2_264961294-264969213 GGATTATAGTAAACCTGATAATCATTATGATGGATTACCAAATATTGAATTGGGAAAATTACTCATACTCTGAATGTTAAAGATGCTGTCCATAAAGATA 4941
Cn_utg2999_44585-52528     GGATTATAGTAAACCTGATAATCATTATGATGGATTACCAAATTTTGAATTAGGAAAATTACTCATACTATGAATGTTAAAGATGCTGTCCATAAAGATA 4930
Cs_LG3_149169260-149177129 GGATTATAGTAAACCTGATAATCATTATGATGGATTACCGAATGTTGAATTGGGTAAATTACTCATACTATGAATGTTAAAGATGCTGTCCATAAAGATA 4858
Cn_utg57260_32948-40788    GGATTATAGTAAACCTGATAATCATTATGATGGATTACCGAATGTTGAATTGGGTATATTACTCATACTATGAATGTTAAAGATGCTGTCCATAAAGATA 4832
                                  5,020               5,040               5,060               5,080               5,100
SbdRT-nis-ori              ATCAATTTTGGAAATTTGGTTTATCTTAATAGTTAGACTAGAGAATGGAAATGAGCTTCTTCAAA-GAGATGAAAAACCCTAGTGGTTTGAATTTCTATG 5050
Cs_LG2_264961294-264969213 ATCAATTTTGGAAATTTGATTTATCTTAATAG---------AGAATGGAAATGAGTTTCTTCAAAAGAGATGAAAAACCCTTGTGGTTTGAATTTCTATG 5032
Cn_utg2999_44585-52528     ATCAATTTTTGAAATTTGGTTTATCTTAATAGTTAGACTAGAGAATGGAAATGAGTTTCTTCAAA-GAGATGAAAAACCCTAGTGGTTTGAATTTCTATG 5029
Cs_LG3_149169260-149177129 ATCAATTTTGGAAATTTGGTTTATCTTAATAGTTAGACTAGAGAATGGAAATGAGTTTCTTCAAA-GAGATGAAAAACCCTAGTGGTTTGAATTTCTATG 4957
Cn_utg57260_32948-40788    ATCAATTTTGGAAATTTGGTT-ATCTTAATAGTTAGACTAGAGAATGGAAATGAGTTTCTTCAAA-GAGATGAAAAACCCTAGTGGTTTGAATTTCTATG 4930
                                  5,120               5,140               5,160               5,180               5,200
SbdRT-nis-ori              TAGAAATCATATGGACAATATTCTACACATATTTAATGCATGTCTTATGACAAGATTATTAATCATACTTAAGCAATTAATTGTGATTGAATATTCTTTT 5150
Cs_LG2_264961294-264969213 TAGAAATCATATGGACAATATTGTACACATATTTAATGCATGTTTCATGACAAGATTATTGATCATACTTAAGCAATTAATTATGATTGAATATTCTTTC 5132
Cn_utg2999_44585-52528     TAGAAATCATATGGACAATATTCTACACATATTTAATGCATGTCTTATGACAAGATTATTAATCATACTTAAGCAATTAATTGTGATTGAATATTCTTTT 5129
Cs_LG3_149169260-149177129 TAGAAATCATATGGACAATATTGTACACATATTTAATGCATGTTTTATGACAAGATTATTAATCATA---------TTAATTGTGATTGAACATTCTTTC 5048
Cn_utg57260_32948-40788    TAGAAATCATATGGACAATATTGTACACATATTTAATGCATGTTTTATGACAAGATTATTAATCATA---------TTAATTGTGATTGAACATTCTTTC 5021
                                  5,220               5,240               5,260               5,280               5,300
SbdRT-nis-ori              CAAGACACATTAAATATATAAAGATGATAATTGTCATACTAAATTTCATTAATATGAAATATGACAATGGAATATCAAAACTCTTTCTTGA-GAAATTAC 5249
Cs_LG2_264961294-264969213 TTAGACACATTAAATATATAAAGATGA-------------------------------------CAATGGAATATCAAAACTCTTTCTTGA-GAAATTAC 5194
Cn_utg2999_44585-52528     CTAGACACATTAAATATATAAAGATGATAATTGTCATACTTGTTTTCATTAATATGAAATATGACAATGGAATATCAAAACTCTTTCTTGA-GAAATTAC 5228
Cs_LG3_149169260-149177129 CTAGACACATTAAATATATAAAGATGATAATTGTCATACTTGTTTTCATTAATATGAAATATGACAATGGAATATCAAAATTCTTTTTTGAAGAAATTAC 5148
Cn_utg57260_32948-40788    CTAAACACATTAAATATATAAAGATGATAATTGTCATACTTGTTTTCATTAATATGAAATATGACAATGGGATATCAAAATTCTTTTTTGAAGAAATTAC 5121
                                  5,320               5,340               5,360               5,380               5,400
SbdRT-nis-ori              TTGAGCGAGGACGGTAAGTAATTGAAAGGTTTTTTGAATTCATATAATCTTTGGAAGTGTACAAAGTGCAATAATTCATGAATGGATAAATGCATGCATC 5349
Cs_LG2_264961294-264969213 TTGAGCGAGGACGGTAAGTAATCGAAAGATTTTT-GAATTCATATAATCTTTGGAAGTGTACGAAGTGCAATAATTAATGAATGGATAAATGCATGCATC 5293
Cn_utg2999_44585-52528     TTGAGCGAGGACGGTAAGTAATCGAAAGGTTTTTTGAATTCATATAATCTTTGGAAGTGTACGAAGTGCAATAATTCATGAATGGATAAATGCATGCATC 5328
Cs_LG3_149169260-149177129 TTGAGCGAGGACGATAAGTAATTGAAAGGTTTTTTGAATTCATATAATCTTTGAAAGTGTACGAAGTGCAATAATTCATGAATGGATAAATGCATGCATC 5248
Cn_utg57260_32948-40788    TTGAGCGAGGACGATAAGTAATTGAAAGGTTTTTTGAATTCATATAATCTTTGAAAGTGTACGAAGTGCAATAATTAATGAATGGATAAATGCATGCATC 5221
                                  5,420               5,440               5,460               5,480               5,500
SbdRT-nis-ori              TATGAAATTATTCTTATATTAAATTATTAAATCC-ATTTTCGATTTATAATTAAAATAAGATAAATTTTAAGTATTCCTGAAAGCTAGTGAGAGTATTAT 5448
Cs_LG2_264961294-264969213 TATAAAATTATTCTTATATTTAATTATTAAATCTTAGTTTTGATTTATAATTGAAATAAGATAAATTTTAAGTATTCCTAAAAGCTAGTGGGAGTATTAT 5393
Cn_utg2999_44585-52528     TATGAAATTATTCTTATGTTAAATTATTAAATCC-ATTTTTGATTTATAATTAAAATAAGATAAATTTTAAGTATTCCTGAAAGCTAGTGGGAGTATTAT 5427
Cs_LG3_149169260-149177129 TATGAAATTATTCTTATGTTTAATTATTAAATCCCATTTTCGATTTATAATTAAAATAAGATAAATTTTAAGTATTCCTGAAAGCTAGTGGGAGTATTAT 5348
Cn_utg57260_32948-40788    TATGAAATTATTCTTATGTTTAATTATTAAATCCCATTTTCGATTTATAATTAAAATAAGATAAATTTTAAGTATTCCTGAAAGCTAGTGGGAGTATTAT 5321
                                  5,520               5,540               5,560               5,580               5,600
SbdRT-nis-ori              GTTAAGATT-TTAAATCTTATATGTTGAATAACATATAGGAAGTAACATTCATTGTAAGCTAAATGTTAAGTCTTAGATTGGAATGAAACTTCACTTATA 5547
Cs_LG2_264961294-264969213 GTTAAGGTT-TTAAATCTTATATGTTGTATAACATATGATAAGTAACATTCCTTGAAAGCTAAATGTTAAGTCTTAGATTGGAATGAAACTTCACTTGTA 5492
Cn_utg2999_44585-52528     GTTAAGGTT-TTAAATCTTATATTTGTATAACATATGAGAAGTAACATTCCTTGAAAGCTAAATGTTAAGTCTTAGATTGGAATGAAACTTCACTTGTA 5526
Cs_LG3_149169260-149177129 GTTAAGTTTCTTAAATCTTATATGTTGTATAACATATAGTAAGTAACATTCCTTGTAAGCTAAATGTTATGTCTTAGATTGTAATGAAACTTCACTTGTA 5448
Cn_utg57260_32948-40788    GTTAAGTTTCTTAAATCTTATATGTTGTATAACATATAGTAAGTAACATTCCTTGTAAGCTAAATGTTATGTCTTAGATTGTAATGAAACTTCACTTGTA 5421
                                  5,620               5,640               5,660               5,680               5,700
SbdRT-nis-ori              AGATTTAAGAATTAAAACATATAACCTTGAAATATATGTTTATTGTTAATAAACATGTGCAGTAAAA-TAAATGATTCTAAATAAAACATGATATATCAT 5646
Cs_LG2_264961294-264969213 AGATTTAAGAATTAAAACATACAACCTTGGAATATATGTTTATTATTAATAAACATGTGCAGTAAAAATAAATGATTCTAA-TAAAACATGATATATCAT 5591
Cn_utg2999_44585-52528     AGATTTAAGAATTAAAACATACAACCTTGGAATATATGTTTATTATTAATAAACATGTGCAGTAAAATTAAATGATTCTAA-TAAAACATGATATATCAT 5625
Cs_LG3_149169260-149177129 AGATTTAAGAATTAAAACATACAACCTTGGAATATATGTTTATTGTTAATAAACATGTGCAGTAAAA-TAAATGATTCTAA-TAAAACATGATATATCAT 5546
Cn_utg57260_32948-40788    AGATTTAAGAATTAAAACATACAACCTTGGAATATATGTTTATTGTTAATAAACATGTGCAGAAAAA-TAAATGATTCTAA-TAAAACATGATATATCAT 5519
                                  5,720               5,740               5,760               5,780               5,800
SbdRT-nis-ori              TTGGGAAATTGATAAATATCTTTTGACAAGATAGAACGTGAATGAATTCTAAGGTTGACCTGTAGATTCATCATGTACATCATGTAGTGTATTAAAATTG 5746
Cs_LG2_264961294-264969213 TTGGGAAATGGATAAATATTTTTTGACAAGATAGAACGTGAATGAAGTCTAAGTTTGACCTGTAGATTCATCATGTACATCATGTAGTGTATTTAAATTG 5691
Cn_utg2999_44585-52528     TTGGGAAATGGATAAATATTTTTTGACAAGATAGAACGTGAATGAAGTCTAAGTTTGACTTGTAGATTCATCATGTACATCATGTTGTGTATTAAAATTG 5725
Cs_LG3_149169260-149177129 TTGGGAAATTGATAAATTTCTTTTGACAAGATAGAACGTGAATGAATTCTAAGGTTGACCTGTAGATTCATCATGTACATCATGTTGTGTATTAGAATTG 5646
Cn_utg57260_32948-40788    TTGGGAAATTGATAAATATCTTTTGACAAGATAGAACGTGAATGAATTCTAAGGTTGACCTGTAGATTCATCATGTACATCATGTTGTGTATTAGAATTG 5619
                                  5,820               5,840               5,860               5,880               5,900
SbdRT-nis-ori              TTGTATCATTCTTAAATGATAGATGATTTGACAACATTGATATCTAATTAAAAGTTATTAGATAACTTGTGTAATGAATATTCTAATAGAATTACATCCT 5846
Cs_LG2_264961294-264969213 TTGTATCATTCTTAAATAAGAAGTGATTTGACAACATTGATATCTAATTAAAAGTTATTAGATAACTTGTGTAATGAATAGTCTAATAGAATTACATCCT 5791
Cn_utg2999_44585-52528     TTGTATCATTCTTGAATAAGAAGTGATTTGACAACATTGATATCTAACTAAAAGTTATTAGATAACTTGTGTAATGAATATTCTAATAGAATTACATCCT 5825
Cs_LG3_149169260-149177129 TTGTATCATTCTTGAATAAGAAGTGATTTGACAACATTGATATCTAACTAAAAGTTATTAGATAACTTGTGTAATGAATATTCTGAAAGAATTACATCCT 5746
Cn_utg57260_32948-40788    TTGTATCATTCTTGAATAAGAAGTGATTTGACAACATTGATATCTAACTAAAAGTTATTAGATAACTTGTGTAATGAATATTCTCAAAGAATTACATCCT 5719
                                  5,920               5,940               5,960               5,980               6,000
SbdRT-nis-ori              AAAA-CCTGGAATTGCTCCTAGATTCTATGGATGAACAATATGTCAAATGTTGTGCAAATTGTCATCAGCTTTAAACTTGAAAGTTAGGAAAGCATCAAA 5945
Cs_LG2_264961294-264969213 TAAAACCTGGAATTGCTCCTAGATTCTATGGATGAACAATATGTCAATTGTTGTGCAAATTGTCATCAGCTTCAAACTTGAAAGTTTGGAAGACATCAAA 5891
Cn_utg2999_44585-52528     CAG--CCTGGAATTGCTCCTAGATTCTATGGATGAACAATATGTCAAATGTTGTGCAAATTGTCATCAGCTTTAAACTTGAAAGTTAGGAAAGCATCAAA 5923
Cs_LG3_149169260-149177129 CAG--CCTGGAATTGCTCCTAGATTCTATGGATGAACAATATGTCAAATGTTGTGCAAATTGTCATCAGCTTTAAACTTGAAAGTTAGGAAAGCATCAAA 5844
Cn_utg57260_32948-40788    CAA--CCTGGAATTGCTCCTAGATTCTATGGATGAACAATATGTCAAATGTTGTGCAAATTGTCATCAACTTTAAACTTGAAAGTTAGGAAAGCATCAAA 5817
```

```
                          6,020 6,040 6,060 6,080 6,100
SbdRT-nis-ori               CATTACACAATAAGATATTAAAGTTTACGGAGTTTTAATGGGCACATTGTTGTTTGTATACAATGATGCTGGTTTAATTGAGATGACACTCAATACTAGT 6045
Cs_LG2_264961294-264969213  CATTACACAATAAGATATTAAAGTTTACGAAGTTTTAATAAGCATATTGTTGTTTGTATACAATGATGCTGGTTTAATTGAGATGACATTCAATACTAGT 5991
Cn_utg2999_44585-52528      CATTACACAATAAGATATTAAAGTTTACGAAGTTTTAATGAGCATATTGTTGTTTGTATACAATGATGCTGGTTTAATTGAGATGACACTCAATACTAGT 6023
Cs_LG3_149169260-149177129  CATTACACAATAAGATATTAAAGTTTACGGAGTTTTAATGAGCATATTGTTGTTTGTATACAATGATGCTGGTTTAATTGAGATGACACTCAATACTAGT 5944
Cn_utg57260_32948-40788     CATTACACAATAAGATATTAAAGTTTACGGAGTTTTAATGAGCATATTGTTGTTTGTATACAATGATGCTGGTTTAATTGAGATGACACTCAATACTAGT 5917

                          6,120 6,140 6,160 6,180 6,200
SbdRT-nis-ori               CATGGTAGAATTTCATCCTTGTTAATGGCCATGTGAACTAGTAGTGCTTATCTAAGAGCACAGTTGCATTATACATTAAAAGAATAAGAATACTTTAAAC 6145
Cs_LG2_264961294-264969213  CGTGGTAGGATTTCATCCTTGTTAATGGCCATGTGATCTAGTTGTGCTTATCTAAGAGCGCAGTTGCATTATACATTAAAAGAATAAGAATACTTTAAAT 6091
Cn_utg2999_44585-52528      CATGGTAGGATTTCATCCTTGTTAATGGCCATGTGAACTAGTTGTGCTAA-CTAAAAGCACAGTTGCATTATACATTAAAAGAATAAGAATACTTTAAAC 6122
Cs_LG3_149169260-149177129  CATGGTAGGATTTCATCCTTGTTAATGGCCATGTGAACTAGTGGTGCTTATCTAAGAGCACAGTTGCATTATACATTAAAAGAATAAGAATACTTTAAAC 6044
Cn_utg57260_32948-40788     CATGGTAGGATTTCATCCTTGTTAATGGCCATGTGAACTAGTGGTGCTTATCTAAGAGCACAGTTGCATTATACATTAAAAGAATAAGAATACTTTAAAC 6017

                          6,220 6,240 6,260 6,280 6,300
SbdRT-nis-ori               TTTAAAAATGTTTGAAAGTTTAAATTGTCATAAATTGGTGATAGAGGATGTTTCACACTTTATGAATTATTATGTGGATTGTTGAATGCACACATACAAG 6245
Cs_LG2_264961294-264969213  TTTAAAA-TGTTTGAAAGTTTAAATTGTCATAAATTGGTGATAGAGGATGTCACACACTTTATGAATTATTATGTGGATTGTTGAATGCACACATACAAG 6190
Cn_utg2999_44585-52528      TTTAAAA-TGTTTGAAAGTTTAAATTGTCATAAATTTGTGATAGAGGATGTTACACACTTTATGAATTATTATGTGAATTGTTGAATGCACACATACAAG 6221
Cs_LG3_149169260-149177129  TTTAAAA-TGTTTGAAAGTTTAAATTGTCATAAATTGGTGATAGAGGATGTCTCACACTTTATGAATTATTATGTGGATTGGTGAATGCACACATACAAG 6143
Cn_utg57260_32948-40788     TTTAAAA-TGTTTGAAAGTTTAAATTGTCATAAATTGGTGATAGAGGATGTCTCACACTTTATGAATTATTATGTGGATTGGTGAATGCACACATACAAG 6116

                          6,320 6,340 6,360 6,380 6,400
SbdRT-nis-ori               GACTTCATTAATTGAATTCTTGAAATAAGTATTCTCAAAATTATGATAGATCAAGTTGGTTGATTTATGCCTTGTCA-ACAAGTATGATTGTCATTACTT 6344
Cs_LG2_264961294-264969213  GACTTCAATAATTGAATTCTTGAAATAAGTATTCTCAAAATTATGATAGATCACGTTGGTTGATTTATGCCTTGTCATACAAGTATGATTGTCATTACTT 6290
Cn_utg2999_44585-52528      GACTTCATTAATTGAATTCTTGAAATAAGTATTCTCAAAATTATGATAGATCACGTTGGTTGATTTATGCATTGTCA-ACAAGTATGATTGTCATTACTT 6320
Cs_LG3_149169260-149177129  GACTTCATTAATTGAATTCTTGAAATAAGTATTCTCAAAATTACGATAGATAAAGTTGGTTAATTTATGCCTTGTCA-ACAAGTATGATTGTTATTACTT 6242
Cn_utg57260_32948-40788     GACTTCATTAATTGAATTCTTGAAATAAGTATTCTCAAAATTACGATAGATCAAGTTGGTTAATTTATGCCTTGTCA-ACAAGTATGATTGTTATTACTT 6215

                          6,420 6,440 6,460 6,480 6,500
SbdRT-nis-ori               GAGGCATAATTCATCAACTGGAATTTTCATGCATTGATCTATTTGGTATTTGGATAAGGGATGTTAAACAGTTGTTATAATA--AATCAAATACTTGGTA 6442
Cs_LG2_264961294-264969213  GAGGCATAATTCATCAACAGGAATTTTCATGCATTGATCTATTTGGTATTTGGATAAGGGATGTTAAACAATTGTTATAATA--AATCAAATACTTGGTA 6388
Cn_utg2999_44585-52528      GAGGCATAATTCATCAACAGGAATTTTCATGCATTGATCTATTTGGTATTTGGATAAGGGATGTTAAACAATTGTTATAATATAAATCAAATACTTGGTA 6420
Cs_LG3_149169260-149177129  GAGGTATAATTCATCAACTGGAATTTTCATGCATTGATCTATTTGGTATTTGGATAAAGGATGTTAAACAATTGTTATAATA--AATCAAATACTAGGTA 6340
Cn_utg57260_32948-40788     GAGGTATAATTCATCAACTGGAATTTTCATGCATTGATCTATTTGGTATTTGGATAAAGGATGTTAAACAATTGTTATAATA--AATCAAATACTAGGTA 6313

                          6,520 6,540 6,560 6,580 6,600
SbdRT-nis-ori               TGTTTGATTTTATAATATATTGTATCCTATATTTGCATGTTTAATCCGTGAATAAATTTATTTATTCTAAATCGTCCGTTGTCAATTAATGATATGGGAG 6542
Cs_LG2_264961294-264969213  TGATTGATTTTATAATATATTGTATCCTATATTTGCATGTTTAATCCGTGAATAAATTTATTTATTCTAAATCATCCGTTGTCGATTAATGATATGGGAG 6488
Cn_utg2999_44585-52528      TGTTTGATTTTATAATATATTGTATCCTATATTTGCATGTTTAATCCGTGAATAAATTTATTTATTCTAAATCGTCCATTGTCGATCAATGATATGGGAG 6520
Cs_LG3_149169260-149177129  TGTTTGATTTTATAGTATATTGTATCCTATATTTGCATGTTTAATCCATGAATAAATTTATTTATTCTAAATCGTCCATTGTCAATTAATGATATGGGAG 6440
Cn_utg57260_32948-40788     TGTTTGATTTTATAATATATTGTATCCTATATTTGCATGTTTAATCCATGAATAAATTTATTTATTCTAAATCGTCCATTGTCAATTAATGATATGGGAG 6413

                          6,620 6,640 6,660 6,680 6,700
SbdRT-nis-ori               TATCATTAAACTAAGACAAATCTGAGAGATATGGTAGAATATACTTGAAAGTATATTGATGAGAATGCATACCATTTTCAGATGATATTCAATGAATTGA 6642
Cs_LG2_264961294-264969213  TATCATTAAACTAAGACAAATCTGAGAGATGTGGTAGAATATACTTGAAAGTATATTGATGAGAATGCATACCAATTTCAGATGATATTCAATGAATTGA 6588
Cn_utg2999_44585-52528      TATCATTAAACTAAGACAAATCTGAGAGATGTGGTAGAATATACTTGAAAGTATATTGATGAGAATGCATACCATTTTTAAATGATATTCAATGAATTGA 6620
Cs_LG3_149169260-149177129  TATCATTAAACTAAGACAAATCTGAGAGATATGGTAGAATGTGCTTGAAAGTATATTGATGAGAATGCATACCAATTTCAGATGATATTCAATGAATTGA 6540
Cn_utg57260_32948-40788     TATCATTAAACTAAGACAAATCTGAGAGATATGGTAGAATGTGCTTGAAAGTATATTGATGAGAATGCATACCAATTTCAGATGATATTCAATGAATTGA 6513

                          6,720 6,740 6,760 6,780 6,800
SbdRT-nis-ori               ATATAGGACTTATCCCAACTACATA--TTACTTTGTGGATATTGTTAT-AAGTAAATTATGAATAGTTATCCTCAGACTTGAGATACCAGATAAGTGTCA 6739
Cs_LG2_264961294-264969213  ATATAGGACTTACCCCAACTACATAATTTACTTTGTGGATATTGTTAT-AAGTAAATTATGAATAGTTATCCTCAGACTTGAGATACCAGATAAGTGTCA 6687
Cn_utg2999_44585-52528      ATATAGGACTTATCCCAACTACATAATT-ACTTTATGGATATTGTTGTTAAGTAAATTATGAATAGTTATCCTCAGACTTGAGATACCAGATAAGTGTCA 6719
Cs_LG3_149169260-149177129  ATATAGGACTTATCCCAACTACAAAATTTACTTTGTGGATATTGTTAT-AAGTAAATTATGAATAGTTATCCTCAGACTTGAGATACCAGATAAGTGTCA 6639
Cn_utg57260_32948-40788     ATATAGGACTTATCCCAACTACAAAATTTACTTTGTGGATATTGTTAT-AAGTAAATTATGAATAGTTATCCTCAGACTTGAGATACCAGATAAGTGTCA 6612

                          6,820 6,840 6,860 6,880 6,900
SbdRT-nis-ori               TGAATGTAGTGCACTTCTGTGGAACAACTTAACGCTATACGTAACTGGTAGTCATAAGGGTTGTTTCCTGAAGTGTTGCAAAGTTCATGGGTTATTCTCG 6839
Cs_LG2_264961294-264969213  TGAATGTAGTGCACTTCTGTGGAACAACTTAACGCTATACGTAACTGGTAGTCATAAGGGTTGTTTCCTGAAGTGTTGCAAAGTTTGTGGGTTATTCTTG 6787
Cn_utg2999_44585-52528      TGAATGTAGTGCACTT----GGAACAACTTAACGCCATA----ATCGGTAGTCATAAGGGTTGTTTCCTGAAGTGTTGCAAAGTTCGTGGGTTATTCTCG 6811
Cs_LG3_149169260-149177129  TGAATGTAGTGCACTTCTGTGGAACAACTTAACGCTATACGTAACTGGTAGTCATAAGGGTTGTTTCCTGAAGTGTTGCAAAGTTCATGGGTTATTCTTG 6739
Cn_utg57260_32948-40788     TGAATGTAGTGCACTTCTGTGGAACAACTTAACGCTATACGTAACTGGTAGTCATAAGGGTTGTTTCCTGAAGTGTTGCAAAGTTCATGGGTTATTCTTG 6712

                          6,920 6,940 6,960 6,980 7,000
SbdRT-nis-ori               TAGTCAAGATAGAATTTGTTCCTTCAAATTTATTTTGGAGTTTAATACTGCTGGGCCCCTCGTAGGGTTGACAAGATGTTTGTGTGGCCACACCCAAAGT 6939
Cs_LG2_264961294-264969213  TAGTCAAGATAGAATTTGTTCCTTCAAATTTATTTTGGAGTTTAATACTGCTGGGCCCCTCGTAGGGTTGACAAGATGTTTGCATGGCCATGCCCAAAGT 6887
Cn_utg2999_44585-52528      TAGTCAAGATAGAATTTGTTCCTTCAAATTTATTTTGGAGTTTAATACTGCTGGGCCCCTCGTAGGGTTGACAAGATGTTTGCATGGCCATGCCCAAAGG 6911
Cs_LG3_149169260-149177129  TAGTCAAGATAGAATTTGTTCTTTCAAATTTATTTTGGAGTTTAATACTGCTGGGTCCCTCGTAGGGTTGACAAGATGTTTGTGTGGCCACGCCCAAAGT 6839
Cn_utg57260_32948-40788     TAGTCAAGATAGAATTTGTTCTTTCAAATTTATTTTGGAGTTTAATACTGCTGGGTCCCTCGTAGGGTTGACAAGATGTTTGCGTGGCCACGCCCAAAGT 6812

                          7,020 7,040 7,060 7,080 7,100
SbdRT-nis-ori               TCTCCCCTGAATATGTTCAGAAGATTTGGTAATCTTGCTCAATCACTTATAACGAGAAACAGAATAGTTGGATAAAGAATGACTTAATTCCATATCTTTA 7039
Cs_LG2_264961294-264969213  TCTCCCCTGAATATGTTCAGAAGATTTGGTAATCTTGTTCAATCACTAATAACGAGAAACAGAATGGTTGAATAAAGAATGACTTAATTCCATATCTTTA 6987
Cn_utg2999_44585-52528      TCTCCCCTGAATATGTTCAGAAGCTTTGGTAATCTTGTTCAATCACTAATAACGAGAAACAGAATAGTTGAATAAAGAATGACTTAATTCCATATCTTTA 7011
Cs_LG3_149169260-149177129  TCTCCCCTGAATATGTTCAGAAGATTTGGTAATCATGCTCAATCACTTATAACGAGAAACAGAATAGTTGGATAAAGAATGACTTAATTCCATATCTTTA 6939
Cn_utg57260_32948-40788     TCTCCCCTGAATATGTTCAGAAGATTTGGTAATCATGCTCAATCACTTATAACAAGAAACAGAATAGTTGGATAAAGAATGACTTAATTCCATATCTTTA 6912

                          7,120 7,140 7,160 7,180 7,200
SbdRT-nis-ori               TTTAACATGGTATTAGAAACAAAAGGATAATAATAATAATGAGAAATCATTAAAAGGTTTTCCAGGAGCCTTGCTCGAGTTGTACTAGAGGCATCGATGT 7139
Cs_LG2_264961294-264969213  TTTAACATGGTATTAGAAACAAAAGGATAATAATAAT---GAGAAATCATTAAAAGGTTTTCCAGGAGCCTTGCTCGAGTTGTACTAGAGGCATCGAAGT 7084
Cn_utg2999_44585-52528      TTTAACATGGTATTAGAAACAAAAGGATAATAATAAT---GAGAAATCATTAAAAGGTTTTCCAGGAGCCTTGCTCGAGTTGTACTAGAGGCATCGAAGT 7108
Cs_LG3_149169260-149177129  TTTAACATGGTATTAGAAACAAAAGGATAATAATAAT---GAGAAATCATTAAAAAGTTTTCCAGGAGCCTTGCTCGAGTTGTACTAGAGGCATCGAAGT 7036
Cn_utg57260_32948-40788     TTTAACATGGTATTAGAAACAAAAGGATAATAATAAT---GAGAAATCATTAAAG-GTTTTCC-GGAGCCTTGCTCGAGTTGTACTAGAGGCATCGAAGT 7007

                          7,220 7,240 7,260 7,280 7,300
SbdRT-nis-ori               GTTGCTAGACGCTAACCGAATGTGATCACTCTATTAAGATATGTCCAAGTGGGAGCTGTTGGTTTTATGGACAA-TAAATTAATAGACGTGTGTTAGGAT 7238
Cs_LG2_264961294-264969213  GTTGCTAGACGCTAACCGAATGTGATCACTCTATTAAGATATGTCCAAGTGGGAGCTGTTGGTTTTATGGACAA-TAAATTAATAGATGTGTGTTAGGAT 7183
Cn_utg2999_44585-52528      GTTGCTAGACGCTAACCGAATGTGATCACTGTATTAAGATATGTCCAAGTGGGAGCTGTTGGTTTTATGGACAAATAAATTAATAGACGTGTGTTAGGAT 7208
Cs_LG3_149169260-149177129  GTTGCTAGACGCTAACCGAATGTGATCACTCTATTAAGATATATCCAAGTGGGAGCTATTGGTTTTATGGATA--TAAATTAGTAGACGTGAGTTAGGAT 7134
Cn_utg57260_32948-40788     GTTGCTAGACGCTAACCGAATGTGATCACTCTATTAAGATATATCCAAGTGGGAGCTGTTGGTTTTATGGATA--TAAATTAGTAGACGTGAGTTAGGAT 7105

                          7,320 7,340 7,360 7,380 7,400
SbdRT-nis-ori               CGAACTCGAGACTTTGGGATTGCACACTTTACGTATGAGAATTTAAAGTTAA-TAATTAAATATAAAATGTTATATTTATTTATTTATTAAACAAAATAA 7337
Cs_LG2_264961294-264969213  CGAACTCGAGACTATGGGATTGCACACTTTACGTATGAGAATTTAAAGTTAA-TAATTAAATATAAA-TGTTATATTTATTTATTTATTAAACGAAATAA 7281
Cn_utg2999_44585-52528      CGAACTCGAGACTATGGGATTGCACACTTTACGTATGAGAATTTAAAGTTAA-TAATTAAATATAAA-TGTTGTATTTATTTATTTATTAAACGAAATAA 7306
Cs_LG3_149169260-149177129  CGAACTCGAGACTATGGGATTGCACACTTTACGTATGAGAATTTAAAGTTAAATAATTAAATATAAA-CGTTGTATTTATTTATTTATTAAACGAAATAA 7233
Cn_utg57260_32948-40788     CGAACTCGAGACTATGGGATTGCACACTTTACGTATGAGAATTTAAAGTTAAATAATTAAATATAAA-CGTTGTATTTATTTATTTATTAAACGAAATAA 7204

                          7,420 7,440 7,460 7,480 7,500
SbdRT-nis-ori               CTAACGGACGTTAAGTAATTAATGTAATGGACTAACGGTCGTTAAATGTTTATGGAAAAGGACTAAATTGCAAAAGTTGGGAAGGTTCCGGAATCCCTTA 7437
Cs_LG2_264961294-264969213  CTAACGGACGTTAAGTAATTAATGTAATGGACTAACGGGTGTTAAAGGTATTTGGAAAAGGACTTAATTGCAAAGATTGGGAAGGTTCCGGAATCCCTAT 7381
Cn_utg2999_44585-52528      CTAACGAACGTTAAGTAATTAATGTAATGGACTAACGGGCGTTAAAGGTATTTGGAAAAGGACTTAATTGCAAAGATTGGGAAGGTTCCGGAATCCCTAT 7406
Cs_LG3_149169260-149177129  CTAACGAACGTTAAGTAATTATTGTAATGGACTAACGGGCGTTAAAGGTATTTGGAAAAGGACTTAATTGCAAAAGTTGGGAAGGTTCCGGAATCCCTAT 7333
Cn_utg57260_32948-40788     CTAACGAACGTTAAGTAATTATTGTAATGGACTAACGGGCGTTAAAGGTATTCGGAAAAGGACTTAATTGCAAAAGTTGGGAAGGTTCCGGAATCCCTAT 7304
```

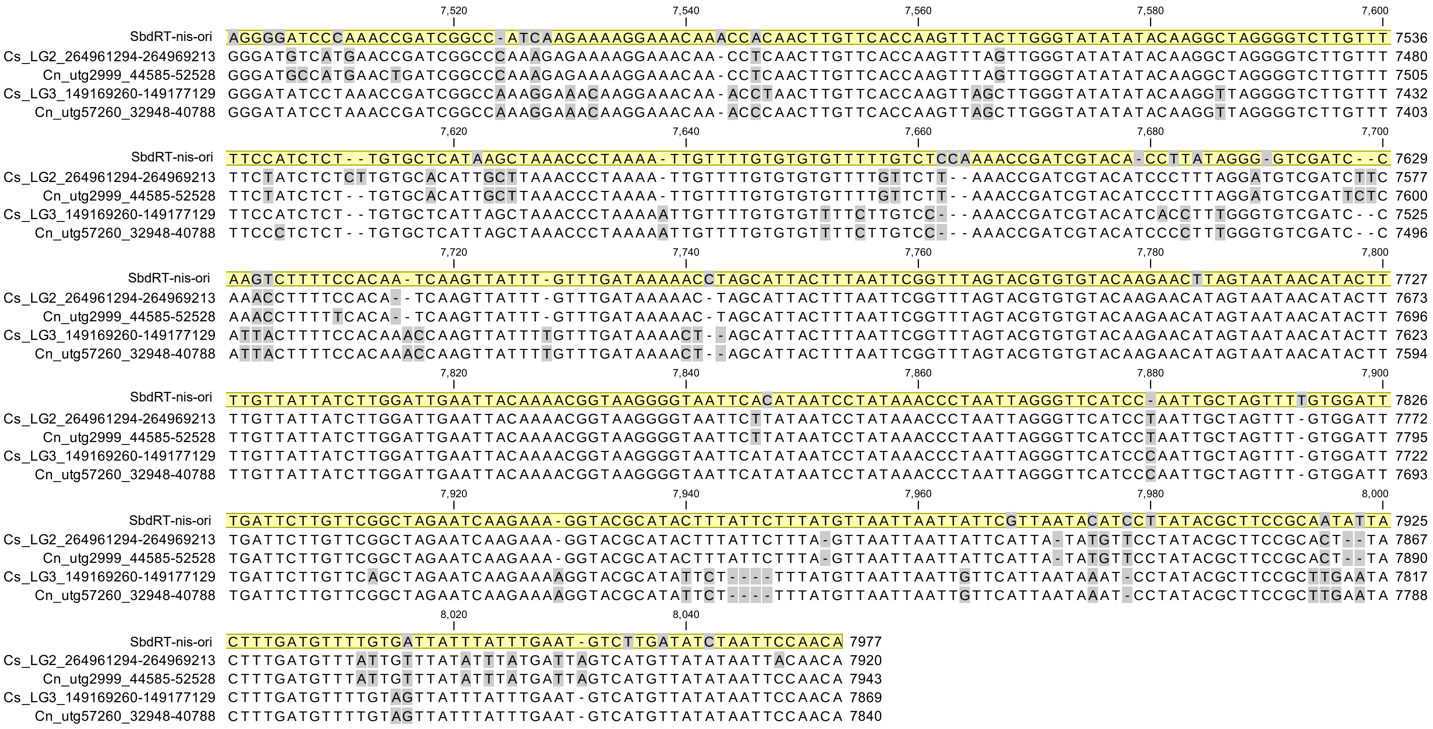

S**upplementary Data 1 | a, The structures of SbdRTs in *C. seticuspe* and *C. nankingense.* a**, DNA sequences of SbdRT-nis-type copies in *C. seticuspe* and *C. nankingense* are aligned. Yellow, pink, and blue boxes represent LTR, PBS-ATG, and NIS, respectively. Cs_LG2_264961294-264969213 and Cs_LG3_1449169269-1449177129 are SbdRT-nis copies in *C. seticuspe.* Cn_utg2999_44585-52528 and Cn_utg57260_32948-40788 are SbdRT-nis copies in *C. nankingense*. Gray regions show sequence differences.

b

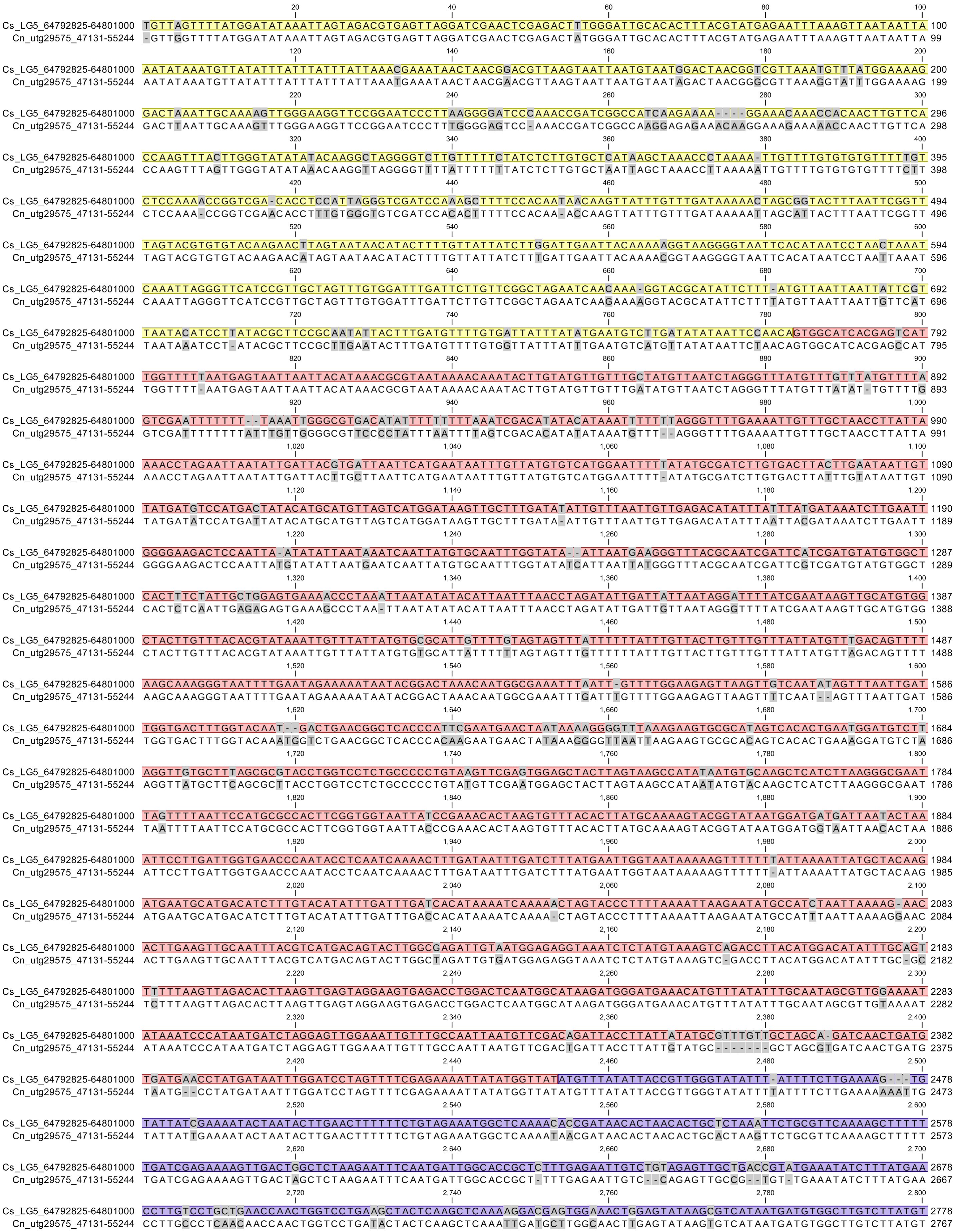

```
                                    2,820               2,840               2,860               2,880               2,900
Cs_LG5_64792825-64801000 TGGGAACAATGGAATCTGCTCTTCAAAAGCAGTTCGAACATTATTTCCCATATAATATGCTCAATGAACTTCGAACTATGTTTGAGAAACCAGCTGCCGT 2878
Cn_utg29575_47131-55244  TGGGAACAATGGAATCTACTCTTCAAAAGCAGTTATAACATTATTTCCCATATAATATGCTCAATGAACTTCGGACTATGTTTGAGAAACCAGCTGCCGT 2867
                                    2,920               2,940               2,960               2,980               3,000
Cs_LG5_64792825-64801000 TGAGATTTATGACTTGACCGACGCGTTACACAGCTGTAGGCAGGGAGAGGGTAAACCCGTAAGTGCTCATGTGCTGGAAATGAAAGGTTACATGGACCAG 2978
Cn_utg29575_47131-55244  CGAGATTTATGACTTGACCGACACGTTACACAGCTGTAGGCAAGGTGAGGGTAAACCCGTAAGTGCTCATGTACTGGAAATGAAAGGTTACATGGACCAG 2967
                                    3,020               3,040               3,060               3,080               3,100
Cs_LG5_64792825-64801000 CTAAAAGCACTTGGCAAAACTTATGAGGATGACATGGCTGTGAATCTGATTAATCGATCCCTTAACAAGGATTTTAATGGTTTTGTCAGAAATTTCAATA 3078
Cn_utg29575_47131-55244  CTCAAAGCACTTGGCAAAACTTATGATGATGACATGGCTGTGAATCTGATTAATCGATCCCTTAACAAGGATTTTAATGATTTTGTCAGGAATTTCAATA 3067
                                    3,120               3,140               3,160               3,180               3,200
Cs_LG5_64792825-64801000 TGCACTGCGTGGGGAAGACAGTTACTGACCTACACGCATTGCTAATTGACTATGAGAAAGGGTTGCCTCAAAAGGTTCCTACCGCCCAGGTACTTGCTAT 3178
Cn_utg29575_47131-55244  TACAC--CGCTGGGAAGACAAGCATCGACCTACACGCATTGCTTATTGATTATGAGAAAGGGTTGCCTCAAAAGGTTCCTACTGCCCAGGTACTTGCTAT 3165
                                    3,220               3,240               3,260               3,280               3,300
Cs_LG5_64792825-64801000 TCAGGGTGGAAGAATTAATAAGCCAAAACCGCAGCGTGCGAACAACAAGAAGGGGAAGGGTAAGGGAAAGGTTGACAAAGGCAAACAGGTGATGGCCTAT 3278
Cn_utg29575_47131-55244  TCAGGGTGGAAGGATTAATAAGCCAAAACCGCATCGTGTGAACAACAAGAAGGGGAAGGGTAAGGGTAAGGTTGACCAAGGCAAACAGGTGATGGCCTAT 3265
                                    3,320               3,340               3,360               3,380               3,400
Cs_LG5_64792825-64801000 CAACCTCAACCCAAGAAGAACCCTACTCCTAGGAAGGATAATCCTAAAAAGGATCAAGCATGCCACCATTGCAAGACAGTTGGGCATTGGAAGAGAAACT 3378
Cn_utg29575_47131-55244  CAACCTCAACCCAAGAAGAACCCTACTCCTAGGAAGTAAAATCCTAAAAAGGATCAAGCATGCCACCACGGTAAGATCGTTGGGAATTGGAAGAGAAACT 3365
                                    3,420               3,440               3,460               3,480               3,500
Cs_LG5_64792825-64801000 GTGCTCTCTATCTTGATGAATTGCGGACAAATAAGATGAA---TGCTGGGTCCTCGGGCGCCGGTTCTTCAGGTAATTTATTTGTCATTGAATTATTTAC 3475
Cn_utg29575_47131-55244  GTGCTCTCTATCTTGATGAATTGCGGGCAAATAAGAAGAAGTATGAAGGGTCCTTGGGCGCCGGTTCTTCTGGTAATTTATTTGTCATTGAATTATTTAC 3465
                                    3,520               3,540               3,560               3,580               3,600
Cs_LG5_64792825-64801000 TCTAAATCATAATAATTCCTGGGTATATGATACGGGCTG----------TGGTATTCACATTTGTAATACACTGCAGGGGTTTAGAGTGGAAAGGAAGCT 3565
Cn_utg29575_47131-55244  TCTAAATCATAATAATTCCTGGGTATATGATACGGGCTGATACGGGCTGTGGTATTCACATTTGTAATACACTGCAGGGGTTTAGAGTGGAAATGAAGCT 3565
                                    3,620               3,640               3,660               3,680               3,700
Cs_LG5_64792825-64801000 TGCCTATGGGGAGCAATATCTGCACGTGGGAAATGGCGCACATGCAGCTGTTGAAGCCATAGGAACGTTTAGTTTAGTATTGCCTTCAGGCTTAGTACTG 3665
Cn_utg29575_47131-55244  TGCCTATGGGGAGCAATATCTG-ACGCGGGAAATGGCGCATGCACAA-----GAAGCCATAGGAACGCTTAGTTTAGTATTGCCTTCGGGCTTAGTATCG 3659
                                    3,720               3,740               3,760               3,780               3,800
Cs_LG5_64792825-64801000 TTATTGAACAATTGTCATTACGCACCTTCTATAATTAGAGGTGTTGTTTCCTTTTCAAGTTTGTTGGATGATGGTTTTACTCATACTATTTCAAGTAATG 3765
Cn_utg29575_47131-55244  TTATTAAACAATTGTCATTACACACCTTCTATAATTAGAGGTGTTGTTTCCTTTC--GCTTTGTTGGATGATGGTTTTACTCATACTATTTCACGTAATG 3757
                                    3,820               3,840               3,860               3,880               3,900
Cs_LG5_64792825-64801000 GAATTGATGTTTCTTTAAATGGTGTATTTTTATTTTAAAGCAATTACTGTGAATGGTGTGTTTGAAATTGATATGATTGGAAATGTATCTAACAATAATAA 3865
Cn_utg29575_47131-55244  GAATTGATGTTTCTTTAAATGGTGTATTTTTATTTTAAAGCAATTACTGTGAATGGTGTGTTTGAAATTGATGTGATTGGAAATGAATCTAACAATAATAA 3857
                                    3,920               3,940               3,960               3,980               4,000
Cs_LG5_64792825-64801000 TAATTCAATGTTTAGCATTAACAAGAAGATAAAGTTAGACTTGAATTCTTCTTATCTTTGGCATTGTCGACTTGCTCATATAGGCAAGGCTCGCATGCAT 3965
Cn_utg29575_47131-55244  TAATTCGATGTTTAGCATTAACAAGAAGATACAGTTAGACTTGAATTCTTCTTATCTTTGGCATTGTAGACTTGCTCATATTGGCAAGGCTCGCATGCAT 3957
                                    4,020               4,040               4,060               4,080               4,100
Cs_LG5_64792825-64801000 AAGCTACAACAAGAAGGGCTTTTGCAAAATATCAATGATGAATCATTTGATAAATGTGAGTCATGCATATCTGGGAAGATGACTAAGAAACCTTTTCATA 4065
Cn_utg29575_47131-55244  AAGCTGCAACAAGAAGGGCTTTTGCAAAATATCAATGATGAATCATTTGATAAATGTGAGTCATGCATATCTGGGAAGATGACGAAGAAACCTTTTCATA 4057
                                    4,120               4,140               4,160               4,180               4,200
Cs_LG5_64792825-64801000 GCAATATGGAGAGGGCTTCTGATCTACTTGGTCTTATTCATACTGATGTGTGTGGACCACTTAGACATGTGTCAAAGAGAGGTGCTAGCTACTTCTTAAC 4165
Cn_utg29575_47131-55244  GCAATATGGAGAGGGCTTCTGATCTACTTGGTCTTATTCATACGGATGTGTGTGGACCACTTAGACATGTGTCAAAGAGAGGTGCTAGCTACTTCTTAAC 4157
                                    4,220               4,240               4,260               4,280               4,300
Cs_LG5_64792825-64801000 CTTTACTGATGATTTCAGCCGTTATGGTTATGTTTATTTACTTAAGCATAAACATGAGGTGTTTGAAACATTTAAGGTTTTCAAAGCTGAAGTAGAACTG 4265
Cn_utg29575_47131-55244  CTTTACTGATGATTTCAGCCGTTATGGTTATGTTTATTTACTTAAGCATAAACATGAGGTGTTTGAAACATTTAAGGTTTTCAAAGCTGAAGTAGAACTG 4257
                                    4,320               4,340               4,360               4,380               4,400
Cs_LG5_64792825-64801000 CAATTGGGAAAGAAGATTAAAGCTCTTCGATCCGATAGAGGAGGCGAGTATCTAAGCCAGAGTTTAAAGATTACTTAGCTGAGAATGGAATTGTCCAGA 4365
Cn_utg29575_47131-55244  CAATTGGGAAAGGAGATTAAAGCTCTTCGATCCGATAGAGGA----AGTATCTAAGCTAAGAGTTTAAAGATTACTTAGTCGAGAATGGAATTGTCCAGA 4353
                                    4,420               4,440               4,460               4,480               4,500
Cs_LG5_64792825-64801000 ATCTCACTTCTCCATATACTCCACAACAAAATGGTGTATCTGAGAGAAGAAATCGTACATTGTTGGACATGATACGATCTATGTTTAATCTTACTACTCT 4465
Cn_utg29575_47131-55244  ATCTCACTTCTCCATATACTCCACAACAAAATGGTGTATCTGAGAGAAGAAATCGTACATTGTTGGACATGATACGATCTATGTTTAATCTTACCACTCT 4453
                                    4,520               4,540               4,560               4,580               4,600
Cs_LG5_64792825-64801000 ACCTTTATCCTTTTGGGATTATGCTTTAGAATCTGCTGTTCGTATTCTAAATATGGTTCCCACAAAGAAGGTTGATAAGACACCATATGAAATATGGCAT 4565
Cn_utg29575_47131-55244  TCCTTTATCCTTTTGGGATTATGCTTTGGAATCAGCTGTTCGCATTCTAAATATGGTTCCCACAAAGAAGGTTGATAAGACACCATATGAAATATGGCAT 4553
                                    4,620               4,640               4,660               4,680               4,700
Cs_LG5_64792825-64801000 GGTAAAGCTCCTAACCTGTCTTATTTAAAGGTCTGGGGTTGCGAGGCGTATGTTAGACGTGACTCAGCAGATAAACTCCAACAAAGATCTGTAAAGTGTA 4665
Cn_utg29575_47131-55244  GGTAAAGCTCCTAACCTGTCTTATTTAAAGGTCTGGGGTTGCGAGGCGTATGTTAGACGTGACTCAGCAGATAAACTCCAACAAAGATCTCAGAAGTGTA 4653
                                    4,720               4,740               4,760               4,780               4,800
Cs_LG5_64792825-64801000 TCTTCGTAGGATACCCTAAGGAAACTATGGGTTACTACTTTTACTATCCAACTGATAATAAAGTAATTGTTGCTAGGTATGGAGATTTTCTTGAAAGAGA 4765
Cn_utg29575_47131-55244  TCTTC--AGGATACCCTAAGGAAACTTTGGGTTACTACTTTAAG-ATCCAACCGAAAATAAAGTAATTGTTGCTAGGTATGGAGATTTTCTTGAAAGAGA 4750
                                    4,820               4,840               4,860               4,880               4,900
Cs_LG5_64792825-64801000 TCTCGTATCTCAAGAATTCAGTGGGAGAGTTTATGATCTGGAAGATGATCATGAAGATGCATTACCTTCTGAAAACACTAACGTACT-TCCGGTAGAACC 4864
Cn_utg29575_47131-55244  TCTCGTATCTCAAGAAT----TGGGAGAGTTTATGATCCGGAAGATGATCAAGAAGATACATTACCTTC--AAAACACTAACGCATTCTCCGGTAGAACC 4844
                                    4,920               4,940               4,960               4,980               5,000
Cs_LG5_64792825-64801000 TGATAGTCTGGGTCCTCCACCAGAAGTTGAAGTGATTCCCGTTCGTAGATCCGTAAGGAATATACGTGCACCTGAACGTCTATGTCTTAACGTAGAGGTT 4964
Cn_utg29575_47131-55244  -GATAGTCCGGGTCCTCCACCGTAAGTTGAAGTGATTCCG--TCAGAGATCCGTAAGGAATACGCGTGCACCTGAACGTCTATGTCTTAACGTAGAGGTT 4941
                                    5,020               5,040               5,060               5,080               5,100
Cs_LG5_64792825-64801000 GAGGAAACTATGTTAGGAGATCTTGGAGAGCCTACTAGTTATAAAGCTGCCATGGCAAGTCCTGACAAGGCTAAATGGCAGGTTGCAATGAATGGGGAAA 5064
Cn_utg29575_47131-55244  GAGGAAACTATGTTAGGAGTTCTTGGAGAGCCTACTAGTTATAAAGCTGCCATGGCAAGTCCTGATAAGGATAAATGGCAAGTTGCAATGAATGGGGAAA 5041
                                    5,120               5,140               5,160               5,180               5,200
Cs_LG5_64792825-64801000 TGCAATCCATGAGAGAAAATGAAGTCTGGGTAGAGGTTGACTTACCTCCAAACGCCTCAGTGGTTGGGAGTAAGTGGCTCTATAAGATTAAGACTGACAT 5164
Cn_utg29575_47131-55244  TGCAATCCATGAGAGAAAATGAAGTCTGGGTAGAGGTTGACTTACCTCCAAACGCCACGGTGGTTGGAAGTAAGTGGCTCTATAAGATTAAGACTGACAT 5141
                                    5,220               5,240               5,260               5,280               5,300
Cs_LG5_64792825-64801000 GGATGGCAATGTACACGTCTATAAAGCTCGTCTAGTTGCGCAAGGTTTTACTCAAACTTATGGCATTGATTATGAGGAGACCTTTTCTCCT-GTAGCAGA 5263
Cn_utg29575_47131-55244  GGATGGCAATGTACATGTCTATAAAGCTCGTCTAGTTGCGCAAGGTTTTACTCAAACTTATGGCATTGATTACGAGGAGACCTTTTCTCCTCAGAATGGA 5241
                                    5,320               5,340               5,360               5,380               5,400
Cs_LG5_64792825-64801000 CATTAGAGCTATTAGAATTCTCATAGCTATAGCAGCATACTATGACTATGAGATATGGCAAATGGATGTCAAAACCGCCTTCTTAAATGGTCGCTTAGAT 5363
Cn_utg29575_47131-55244  CATTAGAGCTATTAGAATTCTCATAGCTATAGCAG-ATACTATGACTATGAGATATGGCAAATAGATGTCAAAACCGC-TTCTTAAATGGTCGCT-AGAT 5338
                                    5,420               5,440               5,460               5,480               5,500
Cs_LG5_64792825-64801000 GAAGACATCTATATGAAGCAACCTGAGGGGTTTGTGAATCCACAATTTCCAAACCGTGTATGCAAATTTCAAAGGTCCATTTATGGATTGAAGCAAGCAT 5463
Cn_utg29575_47131-55244  GAAGACATCTATATGAAGCAACCTGAGGGGTTCGTGAATCCACAATT---AAACCGTGTATGCAAACTTCAAAGGTCCATTTATGGATTGAAGCAAGCAT 5435
                                    5,520               5,540               5,560               5,580               5,600
Cs_LG5_64792825-64801000 CAAGGCAATGGAATAAAAGATTTGATGAAGAAATCAAAAGGTATGGATTTGTTCAAAATCTTGATGAGCCATGTGTATATCACAAAGTTAGTGGGAGTGA 5563
Cn_utg29575_47131-55244  CAAGGCAATGGAATAAAAGATTTGATGAAGAAATCAAAAAGTATGGATTTATTCAAAATCTTGATGAGCCATGTGTATATCACAAAGTTAGTGGGAGTGA 5535
```

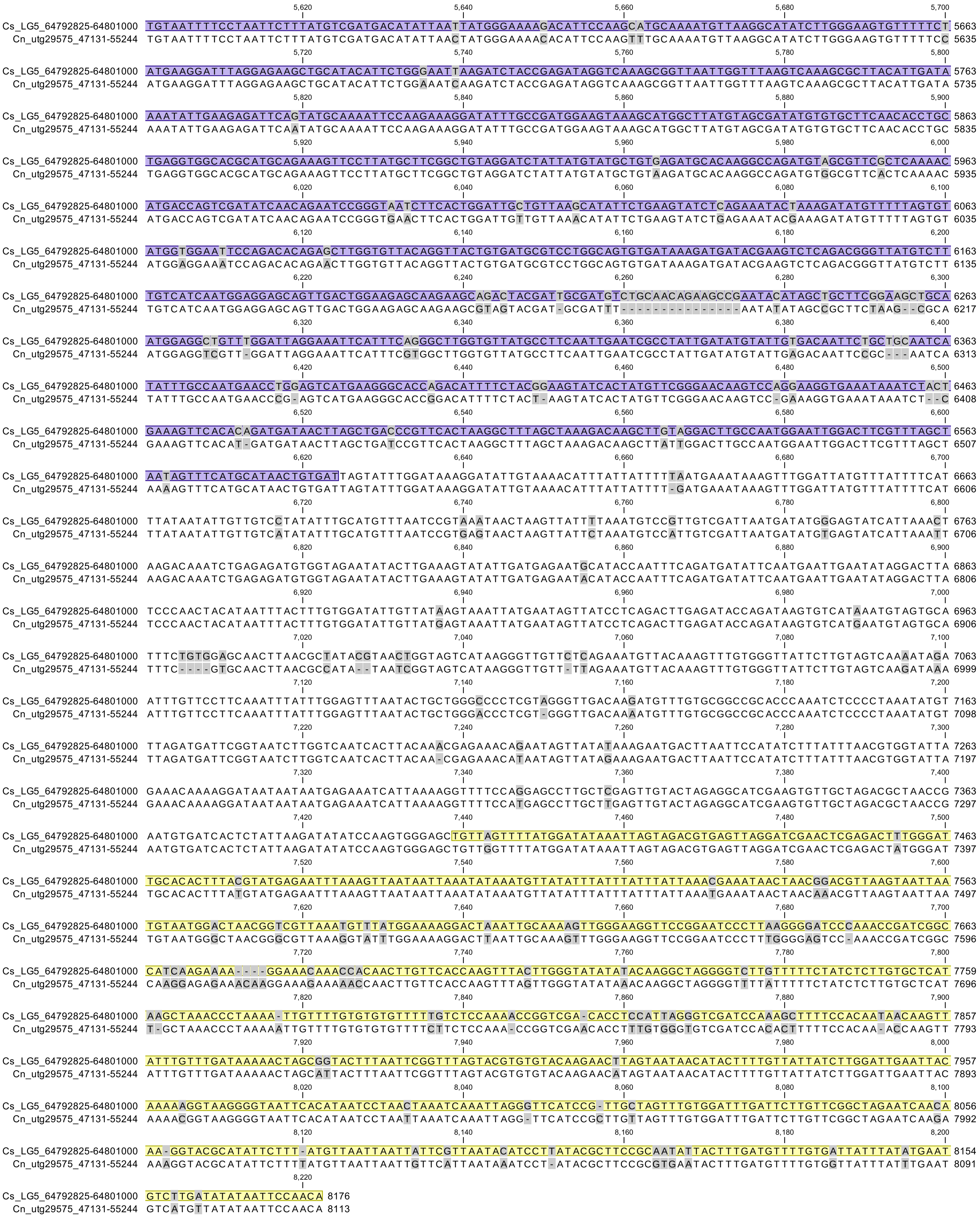

b, DNA sequences of SbdRT-orf-type copies in *C. seticuspe* and *C. nankingense* are aligned. Yellow, pink, and blue boxes indicate LTR, PBS-ATG, and ORF, respectively. Cs_LG5_64792825-64801000 and Cn_utg29575_47131-55244 are SbdRT-orf in *C. seticuspe* and *C. nankingense,* respectively.
