# Supplementary material for: A chromosome-level genome sequence of Chrysanthemum seticuspe, a model species for hexaploid cultivated chrysanthemum
Source: Commun Biol. 2021 Oct 7;4:1167. doi: 10.1038/s42003-021-02704-y (PMC8497461; doi:10.1038/s42003-021-02704-y)
Supplement: Supplementary file 11 — Reporting summary [file 42003_2021_2704_MOESM11_ESM.pdf]

nature research

Corresponding author(s): Makoto Kusaba

Last updated by author(s): Aug 12, 2021

Reporting Summary

Nature Research wishes to improve the reproducibility of the work that we publish. This form provides structure for consistency and transparency in reporting. For further information on Nature Research policies, see our Editorial Policies and the Editorial Policy Checklist.

Statistics

For all statistical analyses, confirm that the following items are present in the figure legend, table legend, main text, or Methods section.

n/a	Confirmed	
☐	☒	The exact sample size (*n*) for each experimental group/condition, given as a discrete number and unit of measurement
☐	☒	A statement on whether measurements were taken from distinct samples or whether the same sample was measured repeatedly
☒	☐	The statistical test(s) used AND whether they are one- or two-sided<br>*Only common tests should be described solely by name; describe more complex techniques in the Methods section.*
☒	☐	A description of all covariates tested
☒	☐	A description of any assumptions or corrections, such as tests of normality and adjustment for multiple comparisons
☐	☒	A full description of the statistical parameters including central tendency (e.g. means) or other basic estimates (e.g. regression coefficient) AND variation (e.g. standard deviation) or associated estimates of uncertainty (e.g. confidence intervals)
☒	☐	For null hypothesis testing, the test statistic (e.g. *F*, *t*, *r*) with confidence intervals, effect sizes, degrees of freedom and *P* value noted<br>*Give P values as exact values whenever suitable.*
☒	☐	For Bayesian analysis, information on the choice of priors and Markov chain Monte Carlo settings
☒	☐	For hierarchical and complex designs, identification of the appropriate level for tests and full reporting of outcomes
☒	☐	Estimates of effect sizes (e.g. Cohen's *d*, Pearson's *r*), indicating how they were calculated

Our web collection on statistics for biologists contains articles on many of the points above.

Software and code

Policy information about availability of computer code

Data collection	Araport11, UniProtKB, Pfam31.0, Repbase v23.05, DANTE tool, timetree, , C. seticuspe (CSE_r1.1), C. nankingense (v2.0), A. annua (Assembly accession: GCA_003112345.1), E. canadensis (V1), sunflower (H. annuus; assembly accession: GCF_002127325.2), lettuce (L. sativa; Assembly accession: GCA_002870075.2), M. micrantha (GCA_009363875.1), coffee (C. canephora; v1.0), grape (V. vinifera ;Assembly version: Genoscope.12X)
Data analysis	Platanus-allee v2.2.2 assembler12, HiRise software pipeline (Dovetail Genomics), SMRT Link v8.0.0.80529, Pilon v1.22, Iso-Seq2 (SMRT Link v5.1), Iso-Seq3 (SMRT Link v6), RepeatModeler v1.0.11, v2.0.1, DIAMOND v0.9.29, RepeatMasker v4.0.7, v4.1.1, BRAKER2 v2.1.5, BLASTP v2.8.0, HMMER v3.2.1, Salmon v1.2.1, GMAP v2020.06.01, BUSCO v4.0.5, MCscanX, R studio v1.4.1106, R v4.0.4, LTR retriever v2.9.0, LTR harvest, LTR Finder, SRA Toolkit v2.9.6, snpEFF 5.0e, KalyoploteR 1.16.0, BLAST+ v2.9.0, CLC main workbench v21.0.2, Orthofinder v2.5.2, MEGA v10.2.4, ClustalW, Samtools 1.12, MAFFT v.7, Trim Galore! v0.6.6, Cutadapt v1.18, HISAT2 v2.1.0, Archaeopteryx.js

For manuscripts utilizing custom algorithms or software that are central to the research but not yet described in published literature, software must be made available to editors and reviewers. We strongly encourage code deposition in a community repository (e.g. GitHub). See the Nature Research guidelines for submitting code & software for further information.

Data

Policy information about availability of data

All manuscripts must include a data availability statement. This statement should provide the following information, where applicable:

- Accession codes, unique identifiers, or web links for publicly available datasets
- A list of figures that have associated raw data
- A description of any restrictions on data availability

Raw datasets were deposited in the DDBJ database under Project ID PRJDB7468 for genomic sequencing and PRJDB5536 for Iso-seq. All sequence datasets, assembled genome sequences, predicted gene models, annotations, and high-quality full-length consensus isoforms are available in PlantGarden (https://plantgarden.jp/)

Field-specific reporting

Please select the one below that is the best fit for your research. If you are not sure, read the appropriate sections before making your selection.

☒ Life sciences ☐ Behavioural & social sciences ☐ Ecological, evolutionary & environmental sciences

For a reference copy of the document with all sections, see nature.com/documents/nr-reporting-summary-flat.pdf

Life sciences study design

All studies must disclose on these points even when the disclosure is negative.

Sample size	For Gojo-0 genome sequence, 317.4 Gb on Hiseq2500 and 343.0 Gb on the PacBio Sequel system were used.
Data exclusions	No data were excluded from the analyses.
Replication	For qRT-PCR analysis, three biological replicates were used.
Randomization	For RT-PCR analysis, samples were randomly chosen.
Blinding	No analysis that requires blinding was performed.

Reporting for specific materials, systems and methods

We require information from authors about some types of materials, experimental systems and methods used in many studies. Here, indicate whether each material, system or method listed is relevant to your study. If you are not sure if a list item applies to your research, read the appropriate section before selecting a response.

Materials & experimental systems

n/a	Involved in the study	
☒	☐	Antibodies
☒	☐	Eukaryotic cell lines
☒	☐	Palaeontology and archaeology
☒	☐	Animals and other organisms
☒	☐	Human research participants
☒	☐	Clinical data
☒	☐	Dual use research of concern

Methods

n/a	Involved in the study	
☒	☐	ChIP-seq
☒	☐	Flow cytometry
☒	☐	MRI-based neuroimaging
